# A hybrid system of mixture models for the prediction of particle size and shape, density, and flowability of pharmaceutical powder blends

**DOI:** 10.1016/j.ijpx.2024.100298

**Published:** 2024-10-28

**Authors:** Mohammad Salehian, Jonathan Moores, Jonathan Goldie, Isra' Ibrahim, Carlota Mendez Torrecillas, Ishwari Wale, Faisal Abbas, Natalie Maclean, John Robertson, Alastair Florence, Daniel Markl

**Affiliations:** a Digital Medicines Manufacturing (DM^2^) Research Centre, Centre for Continuous Manufacturing and Advanced Crystallisation (CMAC), Strathclyde Institute of Pharmacy & Biomedical Sciences, University of Strathclyde, Glasgow, UK; b Centre for Continuous Manufacturing and Advanced Crystallisation (CMAC), Strathclyde Institute of Pharmacy & Biomedical Sciences, University of Strathclyde, Glasgow, UK

**Keywords:** Computational model, Pharmaceutical mixtures, Particle size, Particle shape, Bulk density, Tapped density, True density, Flowability

## Abstract

This paper presents a system of hybrid models that combine both mechanistic and data-driven approaches to predict physical powder blend properties from their raw component properties. Mechanistic, probabilistic models were developed to predict the particle size and shape, represented by aspect ratio, distributions of pharmaceutical blends using those of the raw components. Additionally, the accuracy of existing mixture rules for predicting the blend's true density and bulk density was assessed. Two data-driven models were developed to estimate the mixture's tapped density and flowability (represented by the flow function coefficient, FFC) using data from 86 mixtures, which utilized the principal components of predicted particle size and shape distributions in combination with the true density, and bulk density as input data, saving time and material by removing the need for resource-intensive shear testing for raw components. A model-based uncertainty quantification technique was designed to analyse the precision of model-predicted FFCs. The proposed particle size and shape mixture models outperformed the existing approach (weighted average of distribution percentiles) in terms of prediction accuracy while providing insights into the full distribution of the mixture. The presented hybrid system of models accurately predicts the mixture properties of different formulations and components with often $R^{2}>0.8$, utilising raw material properties to reduce time and material resources on preparing and characterising blends.

## Graphical abstract

**Figure:**
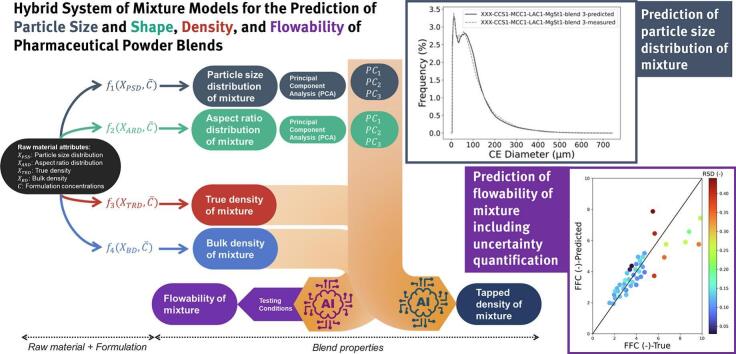

## 1 Introduction

The development of a new drug product involves a series of critical decisions to transform an active pharmaceutical ingredient (API) into a formulated drug product. The extensive research and development process aims to generate the knowledge to make well-informed decisions about process configuration, formulation, operational process conditions and quality control strategy (Kapoor et al., 2021). Traditionally, this process has been sequential, inflexible, and required time-consuming and resource-intensive experimental work to develop the understanding of interactions between raw material properties, process settings, product attributes and environmental factors and their impact on manufacturability, performance, and stability of the final product.

Developing an oral solid dosage form that meets the specifications and can be manufactured at scale requires an appropriate selection of the formulation, manufacturing route and process conditions. In order to achieve manufacturability of the final dosage form, it is necessary for the raw materials to form uniform blends, which exhibit good flow properties without adhering to surfaces and segregation, and allow for compaction into tablets or filling into capsules (Leane et al., 2015). Hence, one crucial factor in the decision-making process is selecting the powder blend's properties and quantifying its impact on manufacturability and the final product quality (Bano et al., 2022a). Particle size and shape distribution, true density, bulk density, tapped density, and flowability are among the key physical powder characteristics that influence the overall process robustness and product quality (White et al., 2022). These physical properties of blends are significantly impacted by the selection the type, grade, and concentrations of excipients, making the formulation design a critical factor.

Historically, formulation settings such as drug loading, excipient selection, and their concentrations have predominantly relied on formulators' experiential knowledge and a trial-and-error approach. This not only results in the consumption of a substantial amount of API, but also leads to prolonged development times and significant resource investment (Sun et al., 2009). The Quality-by-Design (QbD) initiative by the FDA emphasises the need for applying more scientific formulation development approaches, among which is the utilisation of computational models and simulation tools to realise a material-sparing and efficient selection of materials and optimisation of the composition (Lionberger, 2008; Ahmed et al., 2022; Food and Administration, 2004). Computational models present an opportunity to reduce the experimental burden and increase the flexibility to adapt to changes in raw material properties, reducing production time, minimising waste, and enhancing the product consistency.

In recent years, there has been a growing interest in developing mixture models using mechanistic, data-driven, or hybrid approaches to predict blend characteristic (Wang et al., 2016; Reynolds et al., 2017; Moreno-Benito et al., 2022; Wadams et al., 2022; White et al., 2022; Matsunami et al., 2023). The modelling approaches used to predict particle size, shape, density, and flowability of mixtures can be categorised into multiple groups: numerical averages, data-driven (AI-based) modelling techniques, and hybrid models. Particle size and shape of raw materials and the mixtures are key parameters that create a significant impact on the physical properties of powders (Silva et al., 2013; Alyami et al., 2017). The shape descriptor of particles, often described by the aspect ratio (AR), has a considerable influence on the surface energy, cohesion, and adhesion of powders, which individually and collectively impact the flowability of the powder. For example, needle-like structures typically show very poor flow properties whilst more spherical particles are commonly free-flowing (Swaminathan and Kildsig, 2002; Shekunov et al., 2007). Most modelling approaches have been developed to estimate characteristic particle size and shape properties of mixtures based on the raw material attributes. The most common approach is the use of numerical averaging to predict the particle size and shape percentiles based on those of the raw components and their concentrations, demonstrated in previous works using statistical scalars such as median (Van der Bilt et al., 1993) or percentiles (Hilden et al., 2012) of particle size distribution (PSD) and aspect ratio distribution (ARD) of the mixtures. However, prediction of only single values such as $D_{10}$, $D_{50}$, and $D_{90}$ of a distribution disregards the full range of particle properties that can be extracted from the whole particle population (Gamble et al., 2023). The accurate measurement and/or prediction of different types of densities are critical to monitor the physical properties of powders, their impact on the tablet mass and tensile strength, as well as dissolution performance (Stranzinger et al., 2021; Dhondt et al., 2022). The true, bulk and tapped densities are commonly considered as manufacturability-critical density values. Numerical averages have been commonly used to predict true density and bulk density of powders. For example, the harmonic mean is widely used to predict the true density (Moreno-Benito et al., 2022) and bulk density (Robinson et al., 2022) of powder mixtures. In the study by Moreno-Benito et al. (2022), a hybrid model was also proposed for mixture bulk density prediction using a geometric average followed by an artificial neural network (ANN). Alshafiee et al. (2019) used a data-driven model based on radial basis function (RBF) network to predict the bulk density of the mixtures. For the tapped density, however, no predictive modelling study has been conducted so far. In addition to the PSD, ARD, and densities, the flow of a powder is an essential consideration for ensuring robust manufacturing processes. Poor powder flowability can cause detrimental issues in powder transfer during downstream processing, arching in hoppers, and poor die fill during tabletting, often linked to the segregation of API and excipient particles, resulting in suboptimal content uniformity and process operation as well as out of specification drug products. One measure of powder flowability is the flow function coefficient (FFC), which can be obtained through shear cell measurements. Several factors influence powder FFC, such as particle size, surface area, surface energy, and electrostatic properties (Yu et al., 2011; Fu et al., 2012; Samiei et al., 2017). However, despite the complexity of prediction of mixture flowability, only a limited number of baseline models such as empirical equations (Barjat et al., 2021) or approaches based on granular Bond number (Giraud et al., 2021) have appeared in the literature (Bano et al., 2022b). Therefore, data-driven approaches are commonly employed to gain a deeper understanding of these factors and their inter-correlations (Fu et al., 2012; Alshafiee et al., 2019; Barjat et al., 2021; Pereira Diaz et al., 2023). Recently, White et al. (2022) comparatively evaluated several numerical average mixture rules, including mass-weighted, particle volume-weighted, bulk volume-weighted, and surface area volume-weighted averages, to estimate the bulk density and flowability of mixtures.

Some mixture models, such as numerical averaging methods for true and bulk density prediction, have proved to work well across a number of materials and formulations. However, there are still gaps in predictive modelling of several other mixture properties: 1) there is a lack of models to predict full PSDs and ARDs of blends; 2) current models for flowability and tapped density have shown limited capabilities in the accurate prediction of mixture characteristics; 3) lack of uncertainty quantification when estimating powder flowability; and 4) limited consideration of domain knowledge in data-driven modelling approaches and taking combinational impact of multiple relevant properties of raw components into account, such as PSD and ARD of the powder. Hence, an integrated mixture modelling workflow does not exist that combines all influential raw component properties to predict blend properties to inform manufacturability assessment of a given formulation.

**Fig. 1:**
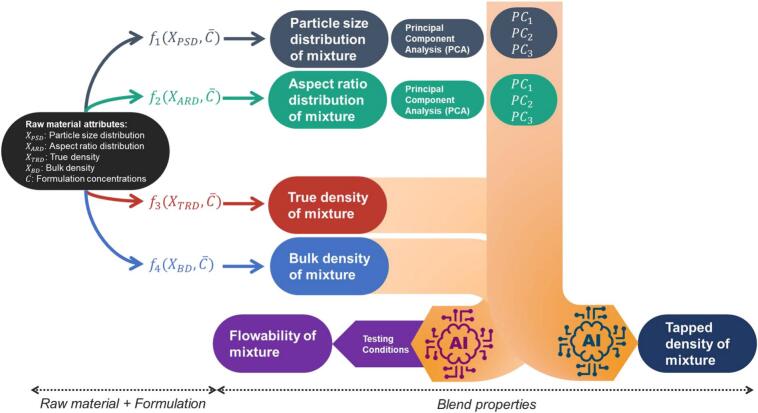
Flow diagram of the proposed hybrid framework for mixture modelling. $f\left(x\right)$ and AI symbols represent the mechanistic and data-driven models, respectively. The testing conditions refers to the consolidation pressure in the flowability measurement.

This paper presents a system of models to predict key mixture characteristics, including a hybrid of mechanistic and data-driven models for particle size, shape, density, and flowability of the mixture based on raw material properties, formulation settings, and testing conditions (Fig. 1). Hybrid modelling enables the incorporation of established, trustworthy models from existing literature while addressing modelling gaps through the development of new analytical and data-driven solutions. Hence, the proposed system of models is a combination of previous literature on true density and bulk density, and proposed models for particle size and shape distribution, tapped density, and flowability. Several numerical averaging methods for true density and bulk density prediction are implemented, compared, and validated against the experimental data. A mechanistic, probabilistic model is developed for particle size and shape distribution by defining the probability of the presence of each size class of a component in the mixture distribution. The proposed model is tested on number-based PSD and ARD and compared to the existing mass-weighted average of percentiles. Principal component analysis (PCA) (Abdi and Williams, 2010) is employed to project the predicted PSDs and ARDs into a few principal components (i.e. PCA scores), the first three of each are selected to describe particle size and shape in lower dimension while preserving the important characteristics of full distributions.

The principal components, along with predicted mixture true and bulk density, are then used in two regression data-driven models to predict tapped density and flowability of the mixture. A customised data pre-processing technique is used to incorporate the domain knowledge into the data-driven model to improve accuracy of prediction. Support Vector Regression (SVM), Random Forests (RF), Gradient Boosting Regression (GBR), and eXtreme Gradient Boosting (XGBoost) are investigated for regression capabilities and their prediction performance is evaluated. This UQ method not only provides a measure of confidence in the predictions but also contributes to the overall interpretability and robustness of the modelling framework.

A notable advantage of here-proposed hybrid modelling approach is its ability to provide meaningful insights to purely data-driven methods, enhancing model interpretability and robustness. By integrating the mechanistic understandings with data-driven techniques, we ensure that the models not only fit the data well but also adhere to established physical/experimental findings. This hybrid approach is a proof-of-concept towards more interpretable and reliable models, capable of addressing complex relationships in pharmaceutical powder characterisation. Moreover, during the data collection, we adhered to FAIR (Findable, Accessible, Interoperable, and Reusable) data principles (Wise et al., 2019; Scheffler et al., 2022) to facilitate the compatibility and interoperability of datasets collected using different devices and across various organisations.

## 2 Materials and methods

### 2.1 Materials

Fifteen pharmaceutical materials that cover a range of excipients and APIs with a broad range of physical properties were studied (Table 1). The excipients used for the development, calibration and validation of the models are all commonly used direct compression and/or capsule filling excipients for oral solid dosage form drug product formulations (Chaudhari and Patil, 2012). The number of excipients gives a wide range of physical properties (Fig. S1 of the support information), chosen to challenge the models and explore a wide knowledge space. These were then blended into 86 different powder mixtures; the full list of these mixtures can be found in Table S1 of the Supporting Information. Each blend is described by a code which includes the abbreviation of individual components (“XXX” denotes either no-API or no second filler in a mixture code). Table 2 summarises the range of drug loadings and physical properties of mixtures based on their API and for all of them.

**Table 1:** List of materials, suppliers, and abbreviations used in this study.

**Material**	**Grade**	**Supplier**	**Abbreviation**	**Reference**
Croscarmellose sodium	AcDiSol	FMC International	CCS1	–
Croscarmellose sodium	Solutab	Roquette	CCS2	–
Dicalcium phosphate anhydrous	Anhydrous Emcompress®	JRS Pharma	DCPA	–
Lactose monohydrate	FastFlo® 316	Foremost Farms USA	LAC1	–
Lactose monohydrate	FastFlo® 316	Kerry, UK	LAC2	Jolliffe et al. (2022)
Magnesium stearate	Hyqual 5712	Mallinckrodt	MgSt1	–
Magnesium stearate	Ligamed MF-2 V	Peter Greven	MgSt2	Jolliffe et al. (2022)
Mannitol	Pearlitol® 200 SD	Roquette	MAN	–
Microctystalline cellulose	Avicel® PH-102	FMC International	MCC1	–
Microcrystalline cellulose	Avicel® PH102	DuPont Nutrition	MCC2	Jolliffe et al. (2022)
Calcium carbonate	–	Merck	CAL	Jolliffe et al. (2022)
Paracetamol	Standard 6375 powder	Mallinckrodt	APAP	Jolliffe et al. (2022)
Mefenamic Acid	–	Sigma Aldrich	MFA	Jolliffe et al. (2022)
Ibuprofen	Ibuprofen 50	BASF	IBU	Jolliffe et al. (2022)
Griseofulvin	–	Molekula	GRF	**–**

**Table 2:** Summary of the range of drug loadings and blend properties based on the API.

	Drug loading (−)		True density ($g/cm^{3}$)		Bulk density ($g/cm^{3}$)		D[v, 0.05] PSD (μm)		S[v, 0.05] ARD (−)	
API	Min.	Max.	Min.	Max.	Min.	Max.	Min.	Max.	Min.	Max.
Placebo	0	0	1.53	1.92	0.35	0.69	78.70	99.74	0.59	0.73
APAP	0.01	0.53	1.40	1.97	0.33	0.66	84.52	120.80	0.56	0.62
IBU	0.05	0.53	1.29	1.53	0.50	0.60	–	–	–	–
MFA	0.05	0.46	1.36	1.50	0.47	0.57	–	–	–	–
GRF	0.01	0.40	1.54	1.57	0.41	0.52	74.94	105	0.61	0.67
Total	0.00	0.53	1.29	1.97	0.33	0.69	74.94	10.80	0.56	0.73

### 2.2 Blends preparation

For each of the 86 formulations in this work, approximately 700 g of blend was prepared. The required weight fraction of disintegrant, filler(s) and API were added to the medium bin blender bucket and mixed for 20 min in a bin blender (Pharmatech AB-015, Pharmatech, Warwickshire, UK), with a blend speed of 20 rpm and an agitator speed of 200 rpm. The weight fraction of lubricant (i.e. magnesium stearate) was then added to the blend and the mixture was blended for a further 5 min under the same conditions. Note that the data used in this paper is generated only for the initial training and validation of the proposed models and does not reflect the deployment procedure.

The quality of the data was rigorously evaluated through several key aspects: 1) A comprehensive Standard Operating Procedure was prepared for every instrument to provide a consistent practice of data collection, e.g. the methodology and measurement settings for particle size and shape distribution. 2) Data completeness was ensured using conventional data generation methods and classic Design of Experiment (DoE) approaches, such as full factorial design. 3) Adequate sample size and data distribution were validated by generating data from a diverse range of blends, illustrated by varying drug loadings and physical properties (Table 2 in the Supporting Information). 4) Data accuracy and consistency were verified through internal consistency checks of similar mixtures and comparisons with model predictions, with expert reviews by technicians, project scientists, and domain experts further ensuring data accuracy.

### 2.3 Characterisation methods

All measurements were carried out in a controlled laboratory at ambient temperatures of 20-25 °C and 40–60 % relative humidity (RH).

#### 2.3.1 Densities

The bulk and tapped densities for all powders, raw or mixture, were determined using a tapping machine (Dual Autotap, Quantrochrome, Boynton Beach, US) following the British Pharmacopeial standard procedure outlined in Appendix XVII S. Known masses of the powders were placed inside a 100 mL and 250 mL graduated cylinder, either 40 g and 100 g respectively or if the powders exceeded the graduation at these masses 20 g and 50 g were used instead. The volume of the powder was read to the nearest graduated unit and used to obtain the bulk density of the powder using the density equation, i.e. $\rho =\frac{m}{V}$, where m is mass (gr), and V is the volume (${\mathrm{cm}}^{3}$).

The true density of the powders was measured using a nitrogen gas pycnometer (MicroUltrapyc 1200e, Quantachrome, Boynton Beach, UK). The gas pycnometer was attached to a controlled water bath to allow a steady temperature of 25 °C for the gas and internal chambers of the device to minimise any influence of temperature on the measurements. Measurements were carried out following the standard procedure outlined in the Appendix XVII K. of the British Pharmacopoeia. The measurements were taken six times and the average value is reported.

#### 2.3.2 Particle size and Shape

The particle size and shape measurements of the powders were carried out using a light microscope that performed an automated raster scan (Morphologi G3 and G4, Malvern Panalytical, Malvern, UK). A dry powder sample was dispersed using the in-built dispersion unit onto a microscope slide, this sample was then raster scanned to measure a large particle population to gain a representative sample, the particle images were processed via the Morphologi Software.

The number-based, and volume-based PSD and ARD data were extracted in 1001 size classes (i.e. $N_{B}=1001$), while the number-based frequency distributions were used within the modelling algorithms. A population threshold of at least 20,000 particles measured was chosen. If a sample measurement was less than this number, then the measurement was repeated until the threshold was met. If multiple measurements were required for a sample the weighted average values for the frequency distributions were used as the inputs to the models. Materials were dispersed using the dry dispersion method, however, dependent on the material, a reduced compressed air dispersal pressure was used to avoid any structural changes in the powders before measurement if powders were known to be sensitive to more energetic dispersal. The magnesium stearate sample population sizes (at least 20,000 particles) were such that any agglomerates captured would have a minimal impact of the overall measurements (Puckhaber et al., 2024). Moreover, using the number-based frequency distribution to develop the particle models minimised the potential impact of agglomeration on the accuracy of measurements.

#### 2.3.3 Flowability

The flowability of the powders was measured experimentally using a ring shear cell test on a powder flow tester (Brookfield PFT, Brookfield Engineering Laboratories, Inc., Middleboro, USA). This test measures the flowability of a ring of powder of known mass based on Jenike's method (Jenike, 1976). A vaned lid is used to test the ring of powder by introducing vertical consolidation pressure varying between 0.79 and 13.26 kPa and then introducing axial shear stress, the torque force required to cause a shear is recorded. These stress recordings calculate a Mohr's circle and the unconfined yield stress of the powder. This process is repeated five times with increasing consolidation pressures and analysed using the Powder Flow Pro software (Brookfield) which runs the equipment. The flow function coefficient (FFC) of the sample was calculated by dividing the unconfined yield stress by the major principal consolidating stress. The average values of three measurements were taken and used as inputs for the models.

### 2.4 Model development

This section describes the mathematical development of mixture models. First, a probabilistic model for PSDs and ARDs of the mixture is explained. Next, analytical mixture models for true density and bulk density of mixture are presented followed by data-driven, predictive models for mixture tapped density and flowability.

#### 2.4.1 Particle size and shape

##### 2.4.1.1 Particle size distribution

The number-based frequency distribution of the powder mixture, $n_{\mathrm{mix}}$, can be represented by a finite parametric mixture model (Wraith et al., 2014):

$$n_{\mathrm{mix},i}=\sum_{j=1}^{K}\lambda_{j}\times n_{j,i}$$

(1) $$i=1,2,\ldots ,N_{B}$$

where K is the number of components in the mixture, $N_{B}$ is the number of size classes, $\lambda_{j}$ is the probability of membership of the jth component ($\sum_{j=1}^{K}\lambda_{j}=1$) in the mixture, and $n_{i}^{j}$ is the frequency of component j in size class i. The probability of membership of each component can be defined as the ratio of the number of particles of that component to the total number of particles in the mixture as follows:

(2) $$\lambda_{j}=\frac{\sum_{i=1}^{N_{B}}n_{j,i}}{\sum_{k=1}^{K}\sum_{i=1}^{N_{B}}n_{k,i}}$$

The number-frequency of particles of component j in Eq. 2 is determined by diving the total volume of particles of component j ($V_{j}$) to the average volume of each particle of component j in size class i (${\bar{V}}_{j,i}$):

(3) $$\sum_{i=1}^{N_{B}}n_{j,i}=\sum_{i}^{N_{B}}\frac{V_{j}}{{\bar{V}}_{j,i}}=\sum_{i}^{N_{B}}\frac{m_{j}/\rho_{t,j}}{\varphi_{j}\times d_{j,i}^{3}}=\frac{M_{j}}{\rho_{j}\varphi_{j}}\sum_{i}^{N_{B}}\frac{1}{d_{j,i}^{3}}$$

where $\rho_{t,j}$ is the true density of component j, $m_{j}$ is the mass of component j,$d_{j,i}\;$is the circular equivalent (CE) diameter of size class i in the distribution of component j, and $\varphi_{j}$ is a correction (shape) factor for component j. The average volume of each particle is calculated assuming that all particles are spherical, however, $\varphi_{j}$ is considered to mitigate this assumption by taking non-spherical shapes into account. Note that $\varphi_{j}$ is an unknown fitting (tuning) parameter that is optimised by minimising the error between measured distributions and model predictions. Expanding Eq. 3 across all components leads to the total number of particles in the mixture:

(4) $$\sum_{j}^{K}\sum_{i}^{N_{B}}n_{j}=\sum_{j}^{K}\sum_{i}^{N_{B}}\frac{m_{j}/\rho_{t,j}}{\varphi_{j}\times d_{j,i}^{3}}=\sum_{j}^{K}\frac{M_{j}}{\rho_{t,j}\;\varphi_{j}}\sum_{i}^{N_{B}}\frac{1}{d_{j,i}^{3}}$$

The mass of component j can be substituted by the mass fraction of component j ($C_{j}$) and the total mass of the powder mixture ($m_{\mathrm{mix}}$) as follows:

(5) $$m_{j}=C_{j}\times m_{\mathrm{mix}}$$

Substituting Eq. (3), (4), (5) in Eq. 2 will give the formula for $\lambda_{j}$ based on C,ρ,φ,and d:

(6) $$\lambda_{j}=\frac{\frac{C_{j}\times m_{\mathrm{mix}}}{\rho_{t,j}\;\varphi_{j}}\sum_{i}^{N_{B}}\frac{1}{d_{j,i}^{3}}}{\sum_{k}^{K}\frac{C_{k}\times m_{\mathrm{mix}}}{\rho_{t,k}\;\varphi_{k}}\sum_{i}^{N_{B}}\frac{1}{d_{k,i}^{3}}}=\frac{\frac{C_{j}}{\rho_{t,j}\;\varphi_{j}}\sum_{i}^{N_{B}}\frac{1}{d_{j,i}^{3}}}{\sum_{k}^{K}\frac{C_{k}}{\rho_{t,k}\;\varphi_{k}}\sum_{i}^{N_{B}}\frac{1}{d_{k,i}^{3}}}$$

Replacing Eq. 6 in Eq. 1 will result in the probabilistic mixture model:

(7) $$n_{\mathrm{mix},i}=\sum_{j=1}^{K}\frac{\frac{C_{j}}{\rho_{t,j}\;\varphi_{j}}\sum_{i}^{N_{B}}\frac{1}{d_{j,i}^{3}}}{\sum_{k}^{K}\frac{C_{k}}{\rho_{t,k}\varphi_{k}}\sum_{i}^{N_{B}}\frac{1}{d_{k,i}^{3}}}\times n_{j,i}$$

A reduced form of Eq. 7 can be obtained by assuming that all components are of identical shape ($\varphi_{m}=\varphi_{n}$ for m,n=1,…,K):

(8) $$n_{\mathrm{mix},i}=\sum_{j=1}^{k}\frac{\frac{C_{j}}{\rho_{t,j}}\sum_{i}^{N_{B}}\frac{1}{d_{j,i}^{3}}}{\sum_{k}^{K}\frac{C_{k}}{\rho_{t,k}}\sum_{i}^{N_{B}}\frac{1}{d_{k,i}^{3}}}\times n_{j,i}$$

The volume-based frequency distribution can be calculated as a product of number-based frequency distribution using the cubic function of size classes:

$$v_{i}=\frac{n_{i}\times d_{i}^{3}}{\sum_{i}^{N_{B}}n_{i}\times d_{i}^{3}}$$

(9) $$i=1,2,\ldots ,N_{B}$$

**Particle shape distribution:** The shape frequency distribution (represented by aspect ratio in this study) of the mixture can be represented by a similar parametric formulation to Eq. 1:

$$\eta_{\mathrm{mix},i}=\sum_{j=1}^{K}\lambda_{j}\times \eta_{j,i}$$

(10) $$i=1,2,\ldots ,N_{B}$$

where $\eta_{j,i}$ denotes the aspect ratio of $i^{\mathrm{th}}$ size class of component j, and other parameters are identical to those of Eq. 1. The probability of membership of each component ($\lambda_{j}$) is described by the ratio of mass concentration to the true density of the component ($\lambda_{j}=\frac{C_{j}}{\rho_{\text{true},j}}$), resulting in the following equation:

(11) $$\eta_{\mathrm{mix},i}=\sum_{j=1}^{K}\frac{C_{j}}{\rho_{t,j}}\times \eta_{j,i}$$

The percentiles ($D_{10}$, $D_{50}$, $D_{90}$) of the estimated PSDs and ARDs – using proposed probabilistic mixture models – are compared with the classic weighted average method, which is the mass-weighted average of percentiles of each component's distribution (i.e. $\eta_{\mathrm{mix},i}=\sum_{j=1}^{K}C_{j}\times n_{j,i}$ and $\eta_{\mathrm{mix},i}=\sum_{j=1}^{K}C_{j}\times \eta_{j,i}$ for PSD and AR, respectively), followed by Hilden et al. (2012).

The particle size and shape are often represented as high-dimensional distribution data, which can be computationally challenging. To simplify the modelling process, principal component analysis (PCA) is employed to reduce the dimensions of the predicted distributions while retaining the properties of the original data (Abdi and Williams, 2010; Bro and Smilde, 2014). By applying PCA to the estimated particle size and shape distributions of the available mixtures, principal scores are obtained and used to describe the particle size and shape of each mixture in subsequent modelling stages for tapped density and flowability prediction.

#### 2.4.2 Densities

##### 2.4.2.1 True density and bulk density

The mixture true density, $\rho_{t,\mathrm{mix}},$ and bulk density, $\rho_{b,\mathrm{mix}}$, are estimated based on the components' mass concentration $C_{j}$, true density $\rho_{j}^{\mathrm{true}}$, and bulk density $\rho_{t,j}$ of component j=1,…,K. The following mass-weighted harmonic and arithmetic rules, respectively, is used in this study to estimate $\rho_{t,\mathrm{mix}}$ and $\rho_{b,\mathrm{mix}}$ following their reasonable accuracy shown by Moreno-Benito et al. (2022) and White et al. (2022):

(12) $$\rho_{t,\mathrm{mix}}=\frac{\sum_{j}^{K}C_{j}}{\sum_{j}^{K}\frac{C_{j}}{\rho_{t,j}}}$$

(13) $$\rho_{b,\mathrm{mix}}=\frac{\sum_{j}^{k}C_{j}\rho_{b,j}}{\sum_{j}^{k}C_{j}}$$

The $\rho_{t,\mathrm{mix}}$ prediction performance of Eq. 12 is compared with those of the mass-weighted arithmetic mean ($\rho_{t,\mathrm{mix}}=\frac{\sum_{j}^{K}C_{j}\;\rho_{t,j}}{\sum_{j}^{K}C_{j}}$) and mass weighted geometric mean ($\rho_{t,\mathrm{mix}}=\exp\left(\frac{\sum_{j}^{K}C_{j}\;\ln\rho_{t,j}}{\sum_{j}^{K}C_{j}}\right)$), followed by a similar comparison for $\rho_{b,\mathrm{mix}}$ estimation between Eq. 13, mass and true density weighted arithmetic mean ($\rho_{b,\mathrm{mix}}=\frac{\sum_{j}^{k}C_{j}\;\rho_{t,j}\rho_{b,j}}{\sum_{j}^{k}C_{j}\rho_{t,j}}$) and particle volume weighted arithmetic mean ($\rho_{b,\mathrm{mix}}=\frac{\sum_{j}^{K}\frac{C_{j}\;\rho_{b,j}}{\rho_{t,j}}}{\frac{\sum_{j}^{K}C_{j}}{\rho_{t,j}}}$).

##### 2.4.2.2 Tapped density

The tapped density of a mixture, $\rho_{\mathrm{tp},\mathrm{mix}}$, is influenced by the bulk density, $\rho_{b,\mathrm{mix}}$, changes during tapping causing a change in particle packing which is further influenced by particle size, shape, and cohesion. To account for these factors, a data-driven approach is used to predict mixture tapped density from material properties of the blend. In this case, the principal scores of mixture particle size and shape distribution (as explained in Section 2.4.1), mixture true density, and mixture bulk density are used as input parameters (also known as “features”) to develop the data-driven model. Four ML-based regression models (XGBoost, GBR, SVM, RF) are comparatively employed to investigate the prediction performance of mixture tapped density, followed by the relative feature importance analysis (Marcílio and Eler, 2020) using the best-performing model. These models are selected due to the simplicity of implementation, computational efficiency, and reasonable accuracy in similar problems (Friedman, 2002; Segal, 2004; Awad and Khanna, 2015; Chen et al., 2015).

#### 2.4.3 Flowability

Following a similar approach to predicting mixture tapped density in Section 2.4.2, four ML-based regression approaches (XGBoost, GBR, SVM, RF) are used to estimate the mixture flowability, which is represented by the FFC. The approach involves using the principal scores of the number-based PSD, mixture true density, mixture bulk density, and consolidation pressure applied to the powder, the latter taking the impact of testing condition into account. After performing feature engineering (Zheng and Casari, 2018) and initial observation of the marginal impact of particle shape (i.e. principal scores of $\eta_{\mathrm{mix}}$) on the estimated flowability, it was excluded from the input parameters. In this study, regression models are preferred over conventional classification approaches (Alshafiee et al., 2019; Valente et al., 2020; Bano et al., 2022a) for one main reasons. A slight error in the prediction of flowability could cause misclassification of the powder mixture, while a numerical prediction of FFC values provides more information on the proximity of each powder to each flow class, enabling a more transparent analysis of the estimated values. The classification performance of the regression models will then be assessed based on the three ranges of interest provided in Table 3. To impose equal importance on cohesive and non-cohesive powders during the training process and consider the domain knowledge based on the powder flow classification, a new approach for transforming the input data is proposed: Eq. 14 is employed to scale the response y (i.e. FFC) from the original domain to [0,1] to improve the prediction by eliminating the problem of different ranges of response variables during the model training process.

(14) $$u=1-2^{-\frac{y}{\beta }}$$

**Table 3:** The number of FFC measurements in each range of interest.

FFC	Powder behaviour	Number of FFC measurements
FFC < 4	Cohesive	84
4 ≤ FFC < 10	Easy-flowing	85
FFC ≥ 10	Free-flowing	66

The threshold parameter β is set to 4 to scale the FFC<4 values (i.e. cohesive powders as categorised in Table 3) to the lower half of the domain (i.e. 0<u<0.5) and the rest of data (i.e. FFC≥4) to the higher half of the domain (i.e. 0.5<u<1).

## 3 Results and discussions

### 3.1 Particle size and aspect ratio distribution mixture model

**Fig. 2:**
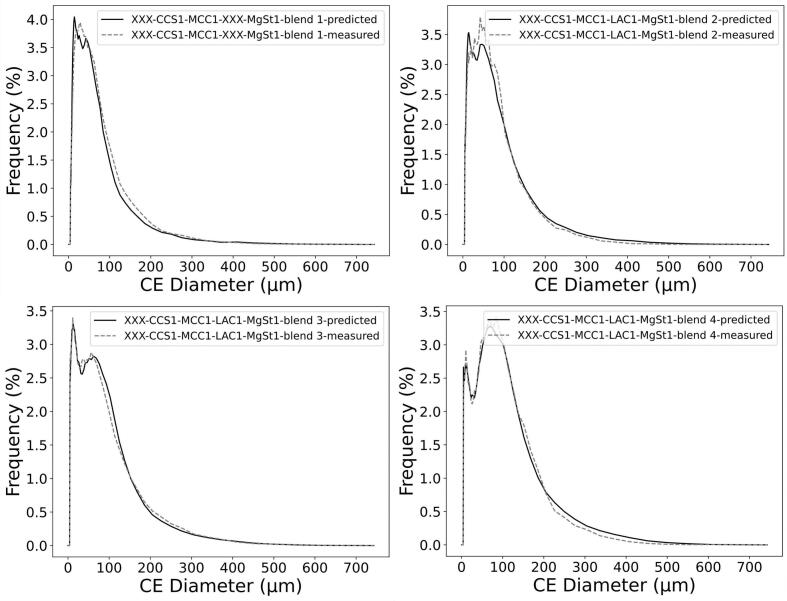
Number-based PSD of mixtures predicted by the probabilistic model vs. experimental measurements for selected formulations. The “XXX” in the blend name denotes no API or no second excipient. Comparisons of other formulations can be found in Fig. S2 in the Supporting Information.

**Fig. 3:**
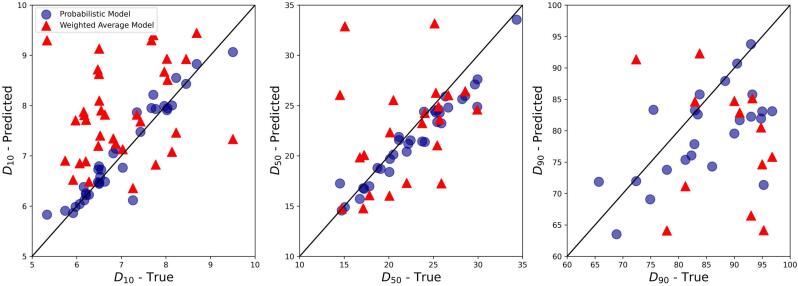
$D_{10}$, $D_{50}$, and $D_{90}$ values of number-based PSDs predicted by probabilistic and weighted average mixture models vs. experimental measurements.

PSD and ARD of 21 mixtures including both placebo (i.e. no API) and APAP mixtures with 5 different excipients (with $N_{B}=1001$) were used to validate the PSD and ARD mixture models. The probabilistic PSD model was applied to all mixtures, showing a reasonable quality of prediction as compared to the measured PSDs (Fig. 2 and Fig. S2 in the Supporting Information). This can be quantitatively demonstrated through the outperformance of the probabilistic mixture model against the classic weighted average method in predicting $D_{10}$, $D_{50}$, and $D_{90}$ of mixture particle size (Fig. 3). For example, the probabilistic model predicted $D_{50}$ of mixture particle size by 74% more accurate than the classic approach. The better prediction performance of the probabilistic approach is shown by its higher correlation of determination ($R^{2}$) and lower root-mean-square error (RMSE) (see Table 4), although the accuracy of both approaches decreased for $D_{90}$ predictions.

**Table 4:** Prediction performance of probabilistic and weighted average mixture models for $D_{10}$, $D_{50}$, and $D_{90}$ of number-based PSDs.

Mixture model	$R^{2}$			RMSE		
	$D_{10}$	$D_{50}$	$D_{90}$	$D_{10}$	$D_{50}$	$D_{90}$
Probabilistic model	0.82	0.78	0.51	0.19	1.75	13.20
Weighted average model	−0.99	−2.32	−2.91	0.65	6.74	4.67

**Fig. 4:**
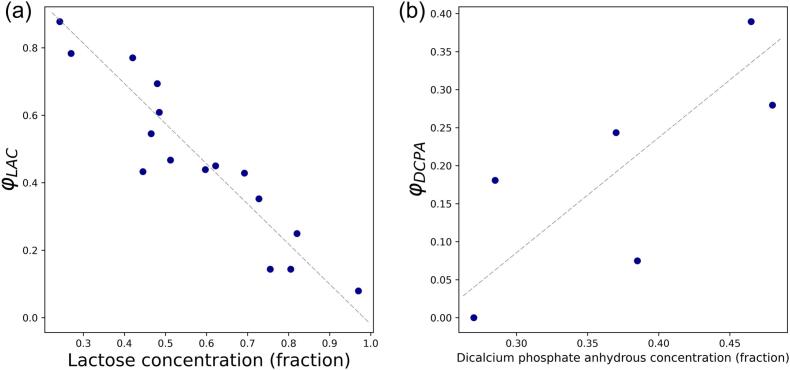
Correction factor of (a) lactose ($\varphi_{\mathrm{LAC}}$) and (b) dicalcium phosphate anhydrous ($\varphi_{\mathrm{DCPA}}$) in probabilistic mixture model vs. their mass concentrations.

A correction factor φ for lactose ($\varphi_{\mathrm{LAC}}$) and dicalcium phosphate anhydrous ($\varphi_{\mathrm{DCPA}}$) is considered to improve the quality of fit of the probabilistic model in Eq. 7. For all other components, φ=1 was considered. The Levenberg-Marquardt algorithm (Roweis, 1996) was employed to optimise $\varphi_{\mathrm{LAC}}$ and $\varphi_{\mathrm{DCPA}}$ for each mixture – if it includes either or both components – through minimising the RMSE between model-predicted PSD and measured data. $\varphi_{\mathrm{LAC}}$ shows a linear correlation with lactose mass concentration, which indicates that the contribution of lactose to the mixture PSD increases with its concentration (Fig. 4a). For DCPA (Fig. 4b), $\varphi_{\mathrm{DCPA}}$ increases with its mass concentration, showing a lower impact on mixture PSD at higher concentrations. In this example, the correction factors are assumed to be independent of formulation to allow the standalone investigation of each component's impact on the PSD of the mixture. The correction factors are calculated to address specific discrepancies between the model predictions and experimental measurements, reflecting the unique physical characteristics of the powders. This has been observed for two excipients, i.e. DCPA and Lactose, that are available in the current dataset. A more in-depth understanding of the impact of physical characteristics of powders on the correction factor requires a multi-variate analysis on a larger dataset with various components. Hence, further studies on the pairwise dependency of components' correction factors can improve the understanding of inter-component interactions.

**Fig. 5:**
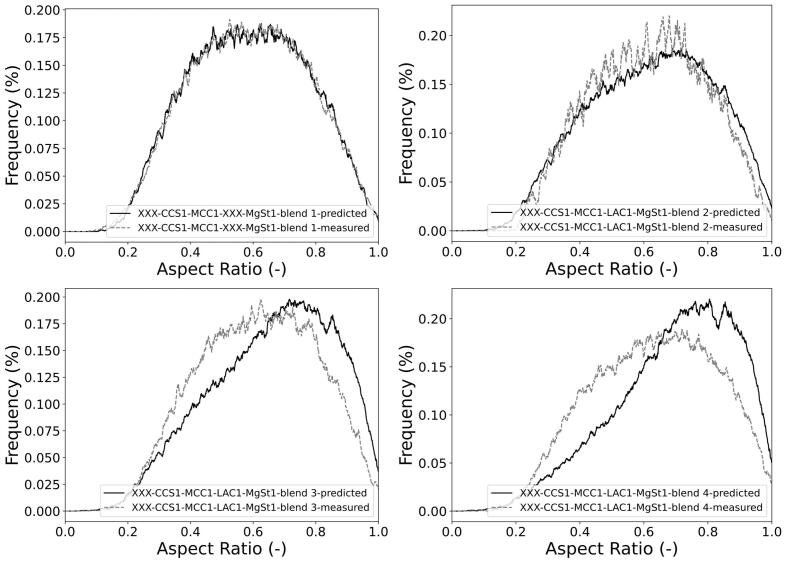
ARDs of mixtures predicted by probabilistic model vs. experimental measurements. The “XXX” in the blend name denotes no API or no second excipient. Comparisons of other formulations can be found in Fig. S3 in the Supporting Information.

**Fig. 6:**
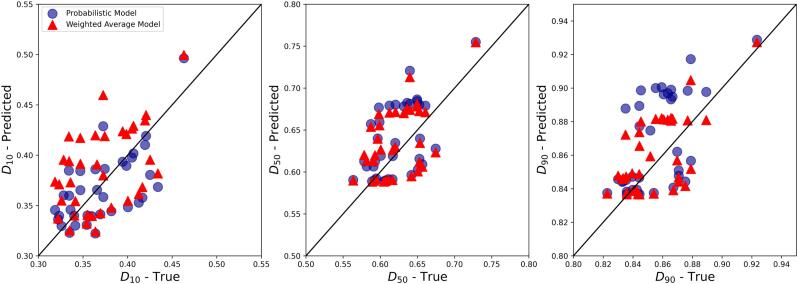
$\;D_{10}$, $D_{50}$, and $D_{90}$ values of ARDs predicted by probabilistic and weighted average mixture models vs. experimental measurements.

Following a similar approach to PSD, the parametric ARD mixture model (Eq. 11) was applied to all mixtures and compared to the measured data (Fig. 5 and Fig. S3 in the Supporting Information). The parametric mixture model outperformed in the estimation of $D_{10}$ and $D_{50}$ of the mixture, whereas the weighted average model resulted in more accurate $D_{90}$ predictions (Fig. 6). The $R^{2}$ and RMSE of both approaches are provided in Table 5.

**Table 5:** Prediction performance of probabilistic and weighted average mixture models for $D_{10}$, $D_{50}$, and $D_{90}$ of number-based ARDs.

Mixture model	$R^{2}$			RMSE		
	$D_{10}$	$D_{50}$	$D_{90}$	$D_{10}$	$D_{50}$	$D_{90}$
Probabilistic model	0.54	0.20	0.20	0.03	0.04	0.03
Weighted average model	−0.08	0.19	0.44	0.05	0.04	0.02

PCA was applied to the set of 35 mixture PSDs and ARDs to project the high dimensional data (35×1001 in this case) onto a few principal components. The explained variance per principal component is evaluated to understand the relative importance of each component to preserve the most important characteristics of the high dimensional data (Fig. S4 in Supporting Information). For PSD (Fig. S4-a) and ARD (Fig. S4-b), the first three components of the PCA models explain 82 % and 94 % of the original data, respectively. The diversity of formulations captured by the PC scores is shown in Fig. S5 in the Supporting Information. To take the effect of particle size and shape into account, these principal components will be used as input features in the data-driven models to predict mixture tapped density and flowability.

### 3.2 True density

**Fig. 7:**
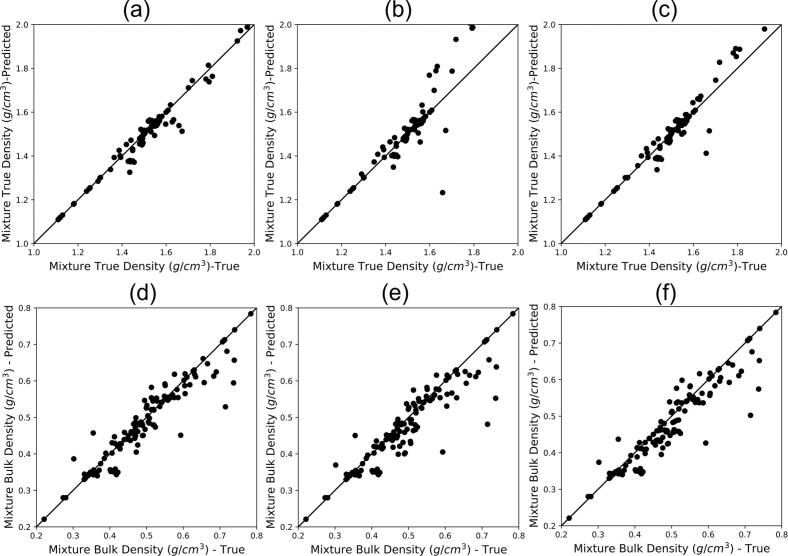
a-c: Predicted mixture true density using (a) mass weight harmonic mean (b) mass weighted arithmetic mean (c) mass weighted geometric mean vs. experimental (true) measurements of blends; d-f: Predicted mixture bulk density using (d) mass-weighted arithmetic mean (e) mass and true density weighted arithmetic mean (f) particle volume weighted arithmetic mean vs. experimental (true) measurements of blends.

Three true density mixture models described in Section 2.4.2 were compared using 86 mixtures (Fig. 7 – a, b, c), the true density of the mixtures varies between 1.11 $g/cm^{3}$ and $1.98\;g/cm^{3}$. The harmonic mean shows the highest accuracy with $R^{2}=0.98$ while the arithmetic and geometric means overestimate mixture true densities above $1.6\;g/cm^{3}$. The outperformance of harmonic mean stems from the mass balance between raw material components and the powder mixture, which requires the reciprocal conversion of true density and using mass fraction to calculate the volume of each component (Eq. 12). Results are in line with the conclusions drawn by Moreno-Benito et al. (2022). A summary of the prediction performance of true density mixture models is provided in Table 6.

**Table 6:** Prediction performance of mixture true density using three mixture rules.

Mixture model	$R^{2}$	RMSE
Harmonic mean	0.98	0.03
Arithmetic mean	0.91	0.08
Geometric mean	0.96	0.05

### 3.3 Bulk density

Similar to Section 3.2, the validity of three bulk density models was assessed using 86 mixture data ranging from 0.22 and 0.87 g/cm^3^. All models displayed similar prediction performance; however, the mass-weighted mixture model exhibited a slightly higher quality of fit ($R^{2}=0.95$) as compared to the mass and true density-weighted and particle volume-weighted approaches (Fig. 7 – d, e, f). Table 7 summarises the prediction performance of mixture rules. In a similar study, White et al. (2022) investigated the accuracy of bulk density mixture models with a dataset varying between 0.1 and 0.4 g/cm^3^ and showed the decrease in models' prediction accuracies for the mixtures with low API bulk densities. This prediction performance degradation, however, was not observed in this study as the dataset here had a wider range of bulk densities.

**Table 7:** Prediction performance of mixture bulk density using three mixture rules.

Mixture model	$R^{2}$	RMSE
Mass weighted mean	0.89	0.04
Mass and true density-weighted mean	0.84	0.05
Particle volume weighted mean	0.86	0.04

### 3.4 Tapped density

**Fig. 8:**
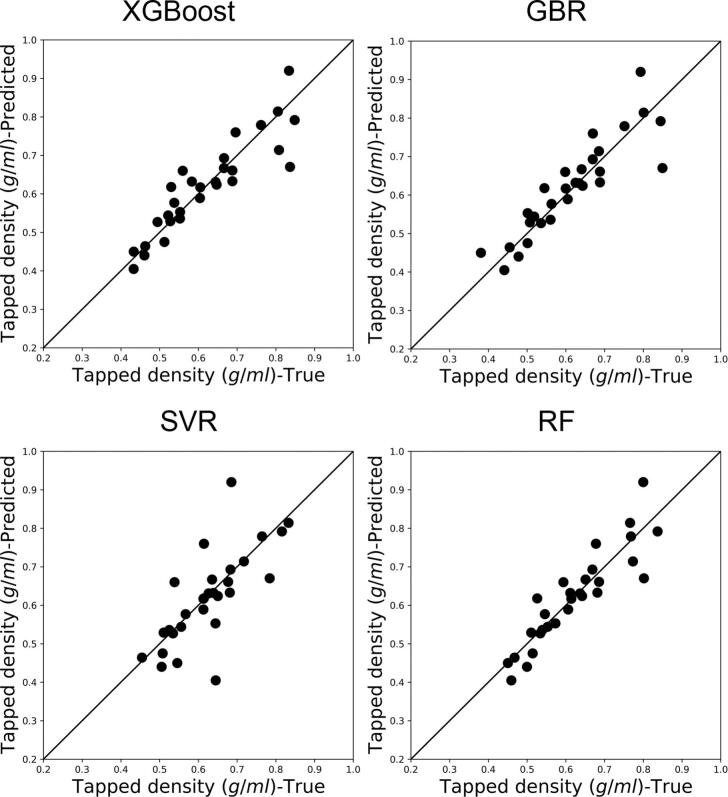
Predicted mixture tapped density using four data-driven models vs. experimental (true) measurements of blends.

The scores of the first three principal components (PC1, PC2, PC3) of mixture PSD, ASD, true density $\rho_{t,\mathrm{mix}}$, and bulk density $\rho_{b,\mathrm{mix}}$ are used as input parameters to train and validate the data-driven tapped density model. These features are used to represent the key material properties and formulation of the mixture, while the model is compatible with other sets of input parameters subject to data availability. This sequential use of the previously developed mixture models' predictions for tapped density estimation preserves the capability of the system model to predict mixture properties using raw material and formulation data. The model was initially trained with 67 training data points, while 30 test data points were kept “unseen” to verify the applicability of the model to new data. For a consistent comparison, the same training and testing procedure was considered for XGBoost, GBR, SVR, and RF. The XGBoost performed slightly better than GBR and RF, while SVR failed to provide a satisfactory prediction performance (Fig. 8). A summary of the prediction performance of tapped density models is provided in Table 8. The feature importance analysis based on XGBoost shows the strong impact of bulk density and particle size on the tapped density of the mixture (Fig. S6 in Supporting Information), corroborating Saw et al. (2013)’s findings on the influence of particle size distribution and tapped density on the bulk density and packing efficiency of milled lactose powders.

**Table 8:** Prediction performance of mixture tapped density using different data-driven models.

Model	$R^{2}$	RMSE
XGBoost	0.80	0.23
GBR	0.78	0.23
SVR	0.51	0.29
RF	0.81	0.22

### 3.5 Flowability

Following a similar approach to tapped density model development (Section 3.4), the first three principal scores (PC1, PC2, PC3) of mixture PSD, mixture true density $\rho_{t,\mathrm{mix}}$, mixture bulk density $\rho_{b,\mathrm{mix}}$ (all describing the material properties of the mixture and predicted by developed models), and consolidation pressure $P_{c}$ (accounting for the process conditions in flowability measurement) are used as input parameters of the data-driven model to predict the FFC of powder mixture. The dataset includes 47 different mixtures, the FFC of which measured under five consolidation pressures, resulting in 235 data points. The train/test splitting was performed based on the mixture formulations to assess the models' capability in estimating new formulations' flowabilities. Four models (as described in Section 2.4.3) were initially trained with 35 mixture data (i.e. 175 data points) while 12 mixtures (i.e. 60 data points) were kept as the test dataset to validate the prediction accuracy of models.

XGBoost and RF both showed higher prediction accuracies ($R^{2}=0.91$) while GBR and SVR, respectively, performed reasonably accurate ($R^{2}=0.86$) and worst ($R^{2}=0.65$), as shown in Table 9 and Fig. S7 in Supporting Information). The powder mixtures were categorised into three groups based on their FFC values (see the highlighted regions in **Error! Reference source not found.** Based on Table 3) to analyse the classification accuracy of the regression models, improving the understanding of predictive models by incorporating the domain knowledge about the often-used flowability classifications (Van Snick et al., 2018; Alshafiee et al., 2019; Valente et al., 2020; Lagare et al., 2023). RF model was selected as the best-performing model due to its high accuracy and general robustness in noisy data as compared to XGBoost (Kirasich et al., 2018). The RF model showed high classification performance with 92% accuracies on test data points (i.e. correct classification of 55 powders out of 60 data points). The model managed to correctly classify all cohesive powders, which possess substantial risk in the manufacturability of the drug product. The confusion matrix in Fig. 10-bottom summarises the overall classification performance of the RF model. To perform further evaluation of the model's predictive performance, the RF model was tested cross-validated using the leave-API-out approach. For each individual APIs that are used in the blend dataset (i.e. 5-fold cross validation based on 5 APIs: placebo, APAP, IBU, MFA, and GRF), a RF model was trained using no instances of the selected API while retraining based on the rest of dataset, and the predictive performance of the trained model for the excluded API was assessed. The RMSEs for the 5-fold cross validated models ranged from 0.098 to 0.196 with an average RMSE of 0.14. As expected, the difference in RMSEs stems from the variability of model's accuracy in predicting the flowability of blends with different APIs. However, the RMSEs from leave-API-out cross-validation are generally lower than the one achieved by the single train-test-split validation (Table 9), showing the robustness of the RF model in predicting the flowability of new APIs. The RF model was also used to predict the FFC of raw material components directly from its raw material characteristics, reducing the need for material to perform flowability measurements (Fig. S8 in Supporting Information).

**Table 9:** Prediction performance of mixture FFC using four data-driven models.

Model	$R^{2}$	RMSE
XGBoost	0.91	0.19
GBR	0.86	0.22
SVR	0.65	0.28
RF	0.91	0.18

**Fig. 9:**
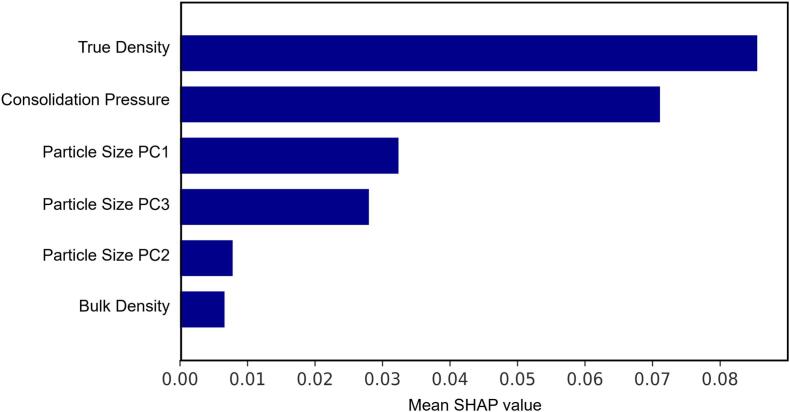
Feature importance analysis for the XGBoost flowability (FFC) mixture model. The features are ranked based on their mean SHAP value.

The mean SHAP values in the RF model were calculated to analyse the predictions from the test dataset (Fig. 9), enhancing the interpretability of the data-driven models and understanding the relative importance of input features. The true density of the mixture, representing the inherent physical property of the material, showed the greatest impact on the predicted FFC, while the high mean SHAP value of consolidation pressure indicates that the model has learned the importance of measurement conditions on mixture flowability. The bulk density is shown to have a minimal impact, which is due to its dependency on particle size, the effect of which is captured by the model. Note that there is an unquantified, yet potentially important effect of uncertainty of the training data on their SHAP relative importance due to the absence of standard deviation measurements in this work. This can be addressed by incorporating the distribution of the input parameters into the training process, such as by including their variance and expected value in the loss function (Shahvandi and Soja, 2022), which is beyond the scope of the current work.

***Uncertainty Quantification (UQ) of FFC predictions:*** Flowability characteristics of the powder are crucial in assessing manufacturability of a given drug blend and formulation, impacting on formulation and process decisions. It is therefore important to determine the precision of powder flow measurements and predictions, particularly when dealing with high FFC values where it is inherently difficult to accurately quantify flow characteristics (Leung et al., 2016). Prediction intervals provide a measure of the uncertainty associated with a predicted value and are used in regression analysis to estimate the range of values within which a future response is expected to fall. Bootstrapping is a statistical resampling technique that is often used as a means of quantifying the uncertainty associated with a machine learning model(Efron, 1992). Classic bootstrapping entails the creation of multiple new datasets by repeatedly sampling, with replacement, from the original training dataset. Each resulting bootstrap sample is subsequently used to train individual regression models. By analysing the variability in their predictions, one can assess confidence intervals and estimate prediction error. An alternative, model-specific approach for estimating prediction error in a RF model leverages the variability in predictions of its constituent decision trees, which are inherently built on resampled datasets derived from the original data. This method capitalises on the intrinsic resampling within the RF algorithm to provide robust error estimates. The prediction error can be calculated based on the assumption that the errors of the regression model are normally distributed. Mathematically, given a new input data point X, the prediction interval (γ) of the predicted response $\hat{y}$ can be obtained as follows:

(15) $$\gamma =\hat{y}\pm Z_{\text{score}}\times E_{s}$$

where $Z_{\text{score}}$ corresponds to the desired level of confidence, and the $E_{s}$ is the standard error calculated as the square root of the sum of the variance of the predicted value and the variance of the residuals. In the case of RF regression, the predicted value is obtained as the mean of the predictions of all trees in the forest (Coulston et al., 2016), and the variance of the predicted value is calculated as the variance of the predictions of all trees in the forest. The variance of the residuals, on the other hand, is estimated as the mean squared error (MSE) of the forest. To obtain $Z_{\text{score}}$, we use the inverse of the cumulative distribution function (CDF) of the standard normal distribution, evaluated at $\left(1-\frac{\alpha }{2}\right)$, where α is the significance level of the confidence interval. In this study, for a 95% prediction interval, α=0.05, and $Z_{\text{score}}$ is the inverse CDF of the standard normal distribution evaluated at $Z_{\text{score}}=0.975$. Given a RF regression model that takes in an input feature vector X and predicts a response value y with a set of n test samples (n=60 in this work), where each sample i has an input feature vector $X_{i}$ and a true response value $y_{i}$. The 95% prediction interval for the predicted response value ${\hat{y}}_{i}$ of a new input feature vector X can be calculated as follows:

(16) $${\hat{y}}_{i}\pm t\left(\frac{\alpha }{2},n-1\right)\times S\times \sqrt{1+\frac{1}{n}+\left(X-\bar{X}\right)\times S_{\mathrm{XX}}^{\mathrm{inv}}\times {\left(X-\bar{X}\right)}^{T}}$$

where:

• ${\hat{y}}_{i}$ is the predicted response value for the input feature vector $X_{i}$
• $t\left(\frac{\alpha }{2},n-1\right)$ is the critical value of the t-distribution with n−1 degrees of freedom and a significance level of $\frac{\alpha }{2}$. In this work, for a 95% prediction interval and n=10, $t\left(0.02559\right)=2.001$ (Dodge, 2008)
• S is the standard deviation of the residuals of the random forest model on the training set
• $\bar{X}$ is the mean of the input feature vectors in the training set
• $S_{\mathrm{XX}}^{\mathrm{inv}}$ is the inverse of the covariance matrix of the input feature vectors in the dataset.

The term $t\left(\frac{\alpha }{2},n-1\right)\times S$ in Eq. 16 accounts for the variability of the model predictions, while the term $\sqrt{1+\frac{1}{n}+\left(X-\bar{X}\right)\times S_{\mathrm{XX}}^{\mathrm{inv}}\times {\left(X-\bar{X}\right)}^{T}}$ is a measure of the variability of the data. The two terms are combined to calculate a prediction interval that captures the uncertainty in both the model and the data. The relative standard deviation (RSD) of the model prediction can be calculated by dividing the prediction interval over the mean predicted response value:

(17) $$\mathrm{RSD}=\frac{2\times t\left(\frac{\alpha }{2},n-1\right)\times S\times \sqrt{1+\frac{1}{n}+\left(X-\bar{X}\right)\times S_{\mathrm{XX}}^{\mathrm{inv}}\times {\left(X-\bar{X}\right)}^{T}}}{{\hat{y}}_{i}}$$

**Fig. 10:**
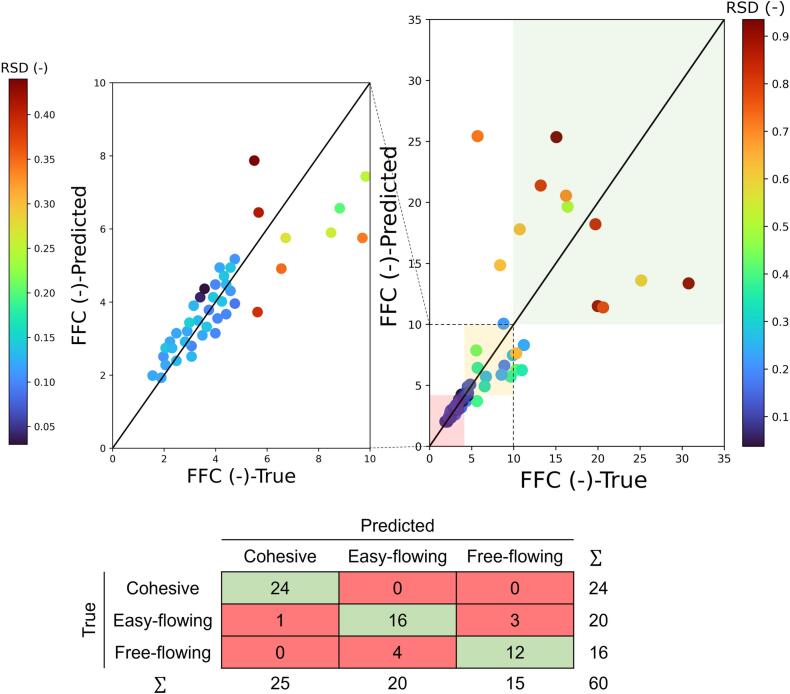
(top) Prediction performance of RF model and the associated uncertainty in predicted FFC values. The colour bar shows the RSD of the predicted data points. (bottom) RF model confusion matrix evaluated on the test dataset.

The uncertainty associated with the input data (X) can be incorporated into the calculation of RSD by modifying $S_{\mathrm{XX}}^{\mathrm{inv}}$. This modification, upon the availability of a statistical variance associated with different measurements, allows for the investigation of how the statistical variance of input features impacts the model's prediction uncertainty. Results show that higher RSD, i.e. greater uncertainty, is associated with the prediction of free-flowing powders with high FFC values (Fig. 10-top). This agrees with the experimental observations of Kuentz and Schirg (2013) and the mathematical description of Leung et al. (2016) who found that the precision of FFC measurement is often compromised by powders with inherently low cohesion values. From the mathematical point of view, the uncertainty of a future prediction is an indicator of the knowledge of the model around that specific data point (Salehian et al., 2022), which explains the higher uncertainty in free-flowing powders of which fewer data points are available in the train/test dataset, i.e. the model has been trained less around the region of feature space with high FFC powder data points. This can be used to modify the target (i.e. objective) function to inform a model-based design of experiments (MBDoE) or model-based optimisation (MBO) approaches, where higher uncertainty of prediction in a single point indicates that the next experiment is likely to be conducted adjacent to that point. It is important to note that data quality has a crucial impact on efficiently achieving the target confidence interval. The quantitative assessment of the relative importance of data quality against the number of data points using various statistical methods, such as sensitivity analysis or Monte Carlo simulation techniques, could be useful.

## 4 Conclusions

A hybrid system of mixture models was presented to predict the key characteristics of pharmaceutical powder blends – focusing on particle size, particle shape, true density, bulk density, tapped density, and flowability – from raw material properties. The proposed system model is built on existing analytical knowledge and complemented with data driven approaches to improve the predictive models' accuracy and shed light on the relationships between blend characteristics and raw material properties. The data-driven models were designed to utilise the outputs from mechanistic models to account for the interplay between material properties while preserving the predictability of the proposed system of models to estimate powder mixture characteristics from raw material properties, hence reducing the cost and material waste during blend preparation and characterisation. The developed ML-based flowability model is of special advantage in predicting the FFC in a fraction of time using a blend's physical properties (e.g. density and particle size/shape), saving material by avoiding the need for comprehensive shear testing of raw materials for flowability measurement. Moreover, the feature importance analysis of the data-driven models showed the agreement between the quantitative (model) and qualitative (domain knowledge) understanding of the influential parameters in the tapped density and flowability of powder mixtures. A new model-based UQ technique was presented to specifically capture the uncertainty associated with the flowability predictions. The proposed model-based UQ strategy can be used for precision analysis of future predictions in development processes and systematic MBDoE.

The models were tested using the experimental data and their accuracy was evaluated across a wide range of formulations from FFC (approximately between 2 and 35), PSD ($D_{50}$ approximately between 15 and 35), ARD ($D_{50}$ approximately between 0.55 and 0.75). The results demonstrate the reliability of the developed system of models in predicting pharmaceutical mixture properties, with their accuracy assessed using several metrics throughout the paper. This system of models holds the potential to accelerate drug product development processes involving new APIs by predicting powder mixture properties, thereby informing formulation and process decisions within a model-based optimisation framework. The leave-API-out cross-validation of the flowability mixture model was performed to prove this potential and assess its predictive performance for new APIs. Future improvements to the robustness of models could include considering further materials (e.g. APIs and excipients with different physical properties), additional important properties of pharmaceutical powders as input parameters (e.g. crystal structure and particle informatics), and expanding the framework by developing new models to predict other characteristics, such as hygroscopicity, permeability, and the effective angle of internal friction. Moreover, the machine learning approaches used in this study can capture the nonlinear behaviour of blend properties at different API concentrations, enabling the investigation of sudden changes of blend properties beyond an API concentration percolation threshold. Acquiring reliable training data, developing and deploying models consistent with mathematical programming and machine learning principles, and regularly maintaining computational frameworks to incorporate new data and enhance their capabilities over time remain as crucial tasks throughout the lifecycle of computational models.

## CRediT authorship contribution statement

**Mohammad Salehian:** Conceptualization, Data curation, Formal analysis, Investigation, Methodology, Software, Validation, Visualization, Writing – original draft. **Jonathan Moores:** Data curation, Methodology, Writing – review & editing. **Jonathan Goldie:** Data curation, Methodology, Writing – review & editing. **Isra' Ibrahim:** Data curation, Writing – review & editing. **Carlota Mendez Torrecillas:** Data curation, Writing – review & editing. **Ishwari Wale:** Data curation, Writing – review & editing. **Faisal Abbas:** Data curation, Writing – review & editing. **Natalie Maclean:** Data curation, Writing – review & editing. **John Robertson:** Funding acquisition, Supervision, Writing – review & editing. **Alastair Florence:** Funding acquisition, Supervision, Writing – review & editing. **Daniel Markl:** Conceptualization, Funding acquisition, Methodology, Project administration, Supervision, Writing – review & editing.

## Declaration of competing interest

The authors declare the following financial interests/personal relationships which may be considered as potential competing interests:

Daniel Markl reports financial support was provided by UK Research and Innovation. Alastair Florence reports financial support was provided by Engineering and Physical Sciences Research Council. If there are other authors, they declare that they have no known competing financial interests or personal relationships that could have appeared to influence the work reported in this paper.

## Data Availability

Data will be made available on request.

## Appendix A Supplementary data

Supplementary material Image 1

## References

[bb0005] Abdi H., Williams L.J. (2010). Principal component analysis. Wiley Interdisc. Rev.: Comp. Stat..

[bb0010] Ahmed K., Pathmanathan P., Kabadi S., Doren J., Kruhlak N., Lumen A., Martinez M., Morrison T., Schuette P., Tegenge M. (2022).

[bb0015] Alshafiee M., AlAlaween W.H., Markl D., Soundaranathan M., Almajaan A., Walton K., Blunt L., Asare-Addo K. (2019). A predictive integrated framework based on the radial basis function for the modelling of the flow of pharmaceutical powders. Int. J. Pharm..

[bb0020] Alyami H., Dahmash E., Bowen J., Mohammed A.R. (2017). An investigation into the effects of excipient particle size, blending techniques and processing parameters on the homogeneity and content uniformity of a blend containing low-dose model drug. PloS One.

[bb0025] Awad M., Khanna R. (2015).

[bb0030] Bano G., Aroniada M., Vueva Y. (2022). 32nd European Symposium on Computer Aided Process Engineering.

[bb0035] Bano G., Aroniada M., Vueva Y. (2022). A model-based approach to predict the flowability of directly compressed pharmaceutical blends from individual components. Computer Aided Chemical Engineering. Elsevier.

[bb0040] Barjat H., Checkley S., Chitu T., Dawson N., Farshchi A., Ferreira A., Gamble J., Leane M., Mitchell A., Morris C. (2021). Demonstration of the feasibility of predicting the flow of pharmaceutically relevant powders from particle and bulk physical properties. J. Pharm. Innov..

[bb0045] Bro R., Smilde A.K. (2014). Principal component analysis. Anal. Methods.

[bb0050] Chaudhari S.P., Patil P.S. (2012). Pharmaceutical excipients: a review. Int J Adv Pharm Biol Chem.

[bb0055] Chen T., He T., Benesty M., Khotilovich V., Tang Y., Cho H., Chen K., Mitchell R., Cano I., Zhou T. (2015). Xgboost: extreme gradient boosting. R package version.

[bb0060] Coulston J.W., Blinn C.E., Thomas V.A., Wynne R.H. (2016). Approximating prediction uncertainty for random forest regression models. Photogramm. Eng. Remote Sens..

[bb0065] Dhondt J., Eeckhout Y., Bertels J., Kumar A., Van Snick B., Klingeleers D., Vervaet C., De Beer T. (2022). A Multivariate Methodology for Material Sparing Characterization and Blend Design in Drug Product Development. Int. J. Pharm..

[bb0070] Dodge Y. (2008).

[bb0075] Efron B. (1992).

[bb0080] Food U.S., Administration Drug (2004).

[bb0085] Friedman J.H. (2002). Stochastic gradient boosting. Computational Statistics and Data Analysis.

[bb0090] Fu X., Huck D., Makein L., Armstrong B., Willen U., Freeman T. (2012). Effect of particle shape and size on flow properties of lactose powders. Particuology.

[bb0095] Gamble J.F., Akseli I., Ferreira A.P., Leane M., Thomas S., Tobyn M., Wadams R.C. (2023). Morphological distribution mapping: Utilisation of modelling to integrate particle size and shape distributions. Int. J. Pharm..

[bb0100] Giraud M., Vaudez S., Gatumel C., Nos J., Gervais T., Bernard-Granger G., Berthiaux H. (2021). Predicting the flowability of powder mixtures from their single components properties through the multi-component population-dependent granular bond number; extension to ground powder mixtures. Powder Technol..

[bb0105] Hilden J., Schrad M., Kuehne-Willmore J., Sloan J. (2012). A first-principles model for prediction of product dose uniformity based on drug substance particle size distribution. J. Pharm. Sci..

[bb0110] Jenike A.W. (1976).

[bb0115] Jolliffe H.G., Ojo E., Mendez C., Houson I., Elkes R., Reynolds G., Kong A., Meehan E., Becker F.A., Piccione P.M. (2022). Linked experimental and modelling approaches for tablet property predictions. Int. J. Pharm..

[bb0120] Kapoor Y., Meyer R.F., Ferguson H.M., Skomski D., Daublain P., Troup G.M., Dalton C., Ramasamy M., Templeton A.C. (2021). Flexibility in Drug Product Development: a Perspective. Mol. Pharm..

[bb0125] Kirasich K., Smith T., Sadler B. (2018). Random forest vs logistic regression: binary classification for heterogeneous datasets. SMU Data Science Review.

[bb0130] Kuentz M., Schirg P. (2013). Powder flow in an automated uniaxial tester and an annular shear cell: a study of pharmaceutical excipients and analytical data comparison. Drug Dev. Ind. Pharm..

[bb0135] Lagare R.B., Huang Y.S., Bush C.O., Young K.L., Rosario A.C.A., Gonzalez M., Mort P., Nagy Z.K., Reklaitis G.V. (2023). Developing a Virtual Flowability Sensor for monitoring a Pharmaceutical Dry Granulation Line. J. Pharm. Sci..

[bb0140] Leane, M., Pitt, K., Reynolds, G., Group, M.C.S.W (2015). A proposal for a drug product Manufacturing Classification System (MCS) for oral solid dosage forms. Pharm. Dev. Technol..

[bb0145] Leung L.Y., Mao C., Chen L.P., Yang C.-Y. (2016). Precision of pharmaceutical powder flow measurement using ring shear tester: High variability is inherent to powders with low cohesion. Powder Technol..

[bb0150] Lionberger R.A. (2008). FDA critical path initiatives: opportunities for generic drug development. AAPS J..

[bb0155] Marcílio W.E., Eler D.M. (2020). From explanations to feature selection: assessing SHAP values as feature selection mechanism, 2020 33rd SIBGRAPI conference on Graphics, patterns and Images (SIBGRAPI). IEEE.

[bb0160] Matsunami K., Miura T., Yaginuma K., Tanabe S., Badr S., Sugiyama H. (2023). Surrogate modeling of dissolution behavior toward efficient design of tablet manufacturing processes. Comput. Chem. Eng..

[bb0165] Moreno-Benito M., Lee K.T., Kaydanov D., Verrier H.M., Blackwood D.O., Doshi P. (2022). Digital twin of a continuous direct compression line for drug product and process design using a hybrid flowsheet modelling approach. Int. J. Pharm..

[bb0170] Pereira Diaz L., Brown C.J., Ojo E., Mustoe C., Florence A.J. (2023). Machine learning approaches to the prediction of powder flow behaviour of pharmaceutical materials from physical properties. Digital Discovery.

[bb0175] Puckhaber D., Finke J.H., David S., Gururajan B., Rane S., Kwade A. (2024). Effect of particle size on the dispersion behavior of magnesium stearate blended with microcrystalline cellulose. Int. J. Pharm..

[bb0180] Reynolds G.K., Campbell J.I., Roberts R.J. (2017). A compressibility based model for predicting the tensile strength of directly compressed pharmaceutical powder mixtures. Int. J. Pharm..

[bb0185] Robinson D.A., Thomas A., Reinsch S., Lebron I., Feeney C.J., Maskell L.C., Wood C.M., Seaton F.M., Emmett B.A., Cosby B.J. (2022). Analytical modelling of soil porosity and bulk density across the soil organic matter and land-use continuum. Sci. Rep..

[bb0190] Roweis S. (1996).

[bb0195] Salehian M., Sefat M.H., Muradov K. (2022). Multi-solution well placement optimization using ensemble learning of surrogate models. J. Petrol. Sci. Eng..

[bb0200] Samiei L., Kelly K., Taylor L., Forbes B., Collins E., Rowland M. (2017). The influence of electrostatic properties on the punch sticking propensity of pharmaceutical blends. Powder Technol..

[bb0205] Scheffler M., Aeschlimann M., Albrecht M., Bereau T., Bungartz H.-J., Felser C., Greiner M., Groß A., Koch C.T., Kremer K. (2022). FAIR data enabling new horizons for materials research. Nature.

[bb0210] Segal M.R. (2004).

[bb0215] Shahvandi M.K., Soja B. (2022). Inclusion of data uncertainty in machine learning and its application in geodetic data science, with case studies for the prediction of Earth orientation parameters and GNSS station coordinate time series. Adv. Space Res..

[bb0220] Shekunov B.Y., Chattopadhyay P., Tong H.H., Chow A.H. (2007). Particle size analysis in pharmaceutics: principles, methods and applications. Pharm. Res..

[bb0225] Silva A.F., Burggraeve A., Denon Q., Van der Meeren P., Sandler N., Van Den Kerkhof T., Hellings M., Vervaet C., Remon J.P., Lopes J.A. (2013). Particle sizing measurements in pharmaceutical applications: Comparison of in-process methods versus off-line methods. Eur. J. Pharm. Biopharm..

[bb0230] Stranzinger S., Markl D., Khinast J., Paudel A. (2021). Review of sensing technologies for measuring powder density variations during pharmaceutical solid dosage form manufacturing. TrAC Trends Anal. Chem..

[bb0235] Sun C.C., Hou H., Gao P., Ma C., Medina C., Alvarez F.J. (2009). Development of a high drug load tablet formulation based on assessment of powder manufacturability: moving towards quality by design. J. Pharm. Sci..

[bb0240] Swaminathan V., Kildsig D.O. (2002). Polydisperse powder mixtures: effect of particle size and shape on mixture stability. Drug Dev. Ind. Pharm..

[bb0245] Saw H.Y., Davies C.E., Paterson A.H., Jones J.R. (2013).

[bb0250] Valente R., Ostapenko A., Sousa B.C., Grubbs J., Massar C.J., Cote D.L., Neamtu R. (2020). Classifying powder flowability for cold spray additive manufacturing using machine learning, 2020 IEEE international conference on big data (big data). IEEE.

[bb0255] Van der Bilt A., Abbink J., Mowlana F., Heath M. (1993). A comparison between data analysis methods concerning particle size distributions obtained by mastication in man. Arch. Oral Biol..

[bb0260] Van Snick B., Dhondt J., Pandelaere K., Bertels J., Mertens R., Klingeleers D., Di Pretoro G., Remon J.P., Vervaet C., De Beer T., Vanhoorne V. (2018). A multivariate raw material property database to facilitate drug product development and enable in-silico design of pharmaceutical dry powder processes. Int. J. Pharm..

[bb0265] Wadams R.C., Akseli I., Albrecht J., Ferreira A.P., Gamble J.F., Leane M., Thomas S., Schuman Y., Taylor L., Tobyn M. (2022). Particle Property Characterization and Data Curation for Effective Powder Property Modeling in the Pharmaceutical Industry. AAPS PharmSciTech.

[bb0270] Wang Y., Snee R.D., Meng W., Muzzio F.J. (2016). Predicting flow behavior of pharmaceutical blends using shear cell methodology: a quality by design approach. Powder Technol..

[bb0275] White L.R., Molloy M., Shaw R.J., Reynolds G.K. (2022). System model driven selection of robust tablet manufacturing processes based on drug loading and formulation physical attributes. Eur. J. Pharm. Sci..

[bb0280] Wise J., de Barron A.G., Splendiani A., Balali-Mood B., Vasant D., Little E., Mellino G., Harrow I., Smith I., Taubert J. (2019). Implementation and relevance of FAIR data principles in biopharmaceutical R&D. Drug Discov. Today.

[bb0285] Wraith D., Mengersen K., Alston C., Rousseau J., Hussein T. (2014).

[bb0290] Yu W., Muteki K., Zhang L., Kim G. (2011). Prediction of bulk powder flow performance using comprehensive particle size and particle shape distributions. J. Pharm. Sci..

[bb0295] Zheng A., Casari A. (2018).

