# Supplementary material for: A hybrid system of mixture models for the prediction of particle size and shape, density, and flowability of pharmaceutical powder blends
Source: Int J Pharm X. 2024 Oct 28;8:100298. doi: 10.1016/j.ijpx.2024.100298 (PMC11584682; doi:10.1016/j.ijpx.2024.100298)
Supplement: Supplementary file 1 — Supplementary material [file mmc1.pdf]

Supporting information for

A Hybrid System of Mixture Models for the Prediction of Particle Size and Shape, Density, and Flowability of Pharmaceutical Powder Blends

Mohammad Salehian[a,b], Jonathan Moores[b], Jonathan Goldie[a,b], Isra' Ibrahim[b], Carlota Mendez Torrecillas[b], Ishwari Wale[b], Faisal Abbas[a,b], Natalie Maclean[b], John Robertson [b], Alastair Florence[a,b], Daniel Markl[a,b*]

[a] *Digital Medicines Manufacturing (DM^2) Research Centre, Centre for Continuous Manufacturing and Advanced Crystallisation (CMAC), Strathclyde Institute of Pharmacy & Biomedical Sciences, University of Strathclyde, Glasgow, UK.*

[b] *Centre for Continuous Manufacturing and Advanced Crystallisation (CMAC), Strathclyde Institute of Pharmacy & Biomedical Sciences, University of Strathclyde, Glasgow, UK.*

**Corresponding author: daniel.markl@strath.ac.uk*

Table S1: Formulations for the powder mixtures.

Mixture code	API	Filler 1	Filler 2	Filler 3	Disintegrant	Lubricant	API conc (w/w)	Filler 1 conc (w/w)	Filler 2 conc (w/w)	Filler 3 conc (w/w)	Disintegrant conc (w/w)	Lubricant conc (w/w)
XXX-MCC1-CCS1-MgSt1- blend 1	---	MCC1	---	---	CCS1	MgSt1	---	0.97	---	---	0.02	0.01
XXX-MCC1-LAC1-CCS1-MgSt1- blend 2	---	MCC1	LAC1	---	CCS1	MgSt1	---	0.7275	0.2425	---	0.02	0.01
XXX-MCC1-LAC1-CCS1-MgSt1- blend 3	---	MCC1	LAC1	---	CCS1	MgSt1	---	0.485	0.4850	---	0.02	0.01
XXX-MCC1-LAC1-CCS1-MgSt1- blend 4	---	MCC1	LAC1	---	CCS1	MgSt1	---	0.2425	0.7275	---	0.02	0.01
XXX-LAC1-CCS1-MgSt1- blend 5	---	LAC1	---	---	CCS1	MgSt1	---	0.9700	---	---	0.02	0.01
APAP-MCC1-CCS1-MgSt1- blend 1	APAP	MCC1	---	---	CCS1	MgSt1	0.01	0.96	---	---	0.02	0.01
APAP-MCC1-CCS1-MgSt1- blend 2	APAP	MCC1	---	---	CCS1	MgSt1	0.2	0.77	---	---	0.02	0.01
APAP-MCC1-CCS1-MgSt1- blend 3	APAP	MCC1	---	---	CCS1	MgSt1	0.4	0.57	---	---	0.02	0.01
APAP-MCC1-LAC1-CCS1-MgSt1- blend 4	APAP	MCC1	LAC1	---	CCS1	MgSt1	0.01	0.48	0.4800	---	0.02	0.01
APAP-MCC1-LAC1-CCS1-MgSt1- blend 5	APAP	MCC1	LAC1	---	CCS1	MgSt1	0.2	0.385	0.3850	---	0.02	0.01
APAP-MCC1-LAC1-CCS1-MgSt1- blend 6	APAP	MCC1	LAC1	---	CCS1	MgSt1	0.4	0.285	0.2850	---	0.02	0.01
APAP-MCC1-CCS1-MgSt1- blend 7	APAP	MCC1	---	---	CCS1	MgSt1	0.01	0.93	---	---	0.05	0.01
APAP-MCC1-CCS1-MgSt1- blend 8	APAP	MCC1	---	---	CCS1	MgSt1	0.1	0.84	---	---	0.05	0.01
APAP-MCC1-CCS1-MgSt1- blend 9	APAP	MCC1	---	---	CCS1	MgSt1	0.4	0.54	---	---	0.05	0.01
APAP-MCC1-LAC1-CCS1-MgSt1- blend 10	APAP	MCC1	LAC1	---	CCS1	MgSt1	0.01	0.465	0.4650	---	0.05	0.01
APAP-MCC1-LAC1-CCS1-MgSt1- blend 11	APAP	MCC1	LAC1	---	CCS1	MgSt1	0.2	0.37	0.3700	---	0.05	0.01
APAP-MCC1-LAC1-CCS1-MgSt1- blend 12	APAP	MCC1	LAC1	---	CCS1	MgSt1	0.4	0.27	0.2700	---	0.05	0.01
APAP-MCC1-CCS1-MgSt1- blend 13	APAP	MCC1	---	---	CCS1	MgSt1	0.01	0.9	---	---	0.08	0.01
APAP-MCC1-CCS1-MgSt1- blend 14	APAP	MCC1	---	---	CCS1	MgSt1	0.2	0.71	---	---	0.08	0.01

APAP-MCC1-CCS1-MgSt1- blend 15	APAP	MCC1	---	---	CCS1	MgSt1	0.4	0.51	---	---	0.08	0.01
APAP-MCC1-LAC1-CCS1-MgSt1- blend 16	APAP	MCC1	LAC1	---	CCS1	MgSt1	0.01	0.45	0.4500	---	0.08	0.01
APAP-MCC1-LAC1-CCS1-MgSt1- blend 17	APAP	MCC1	LAC1	---	CCS1	MgSt1	0.2	0.355	0.3550	---	0.08	0.01
APAP-MCC1-LAC1-CCS1-MgSt1- blend 18	APAP	MCC1	LAC1	---	CCS1	MgSt1	0.4	0.255	0.2550	---	0.08	0.01
XXX-MCC1-CCS2-MgSt2- blend 1	---	MCC1	---	---	CCS2	MgSt2	0	0.816	0	---	0.143	0.041
XXX-LAC2-CAL- blend 1	---	LAC2	CAL	---	---	---	0	0.934	0.066	---	0	0
XXX-LAC2-CAL- blend 2	---	LAC2	CAL	---	---	---	0	0.735	0.265	---	0	0
XXX-LAC2-CAL- blend 3	---	LAC2	CAL	---	---	---	0	0.47	0.53	---	0	0
XXX-LAC2-MCC1-CAL-CCS2-MgSt2- blend 1	---	LAC2	MCC1	CAL	CCS2	MgSt2	0	0.705	0.2	0.05	0.035	0.01
XXX-LAC2-MCC1-CAL-CCS2-MgSt2- blend 2	---	LAC2	MCC1	CAL	CCS2	MgSt2	0	0.555	0.2	0.2	0.035	0.01
APAP-LAC2- blend 1	APAP	LAC2	---	---	---	---	0.133	0.868	0	---	0	0
APAP-LAC2- blend 2	APAP	LAC2	---	---	---	---	0.265	0.735	0	---	0	0.00
APAP-LAC2- blend 3	APAP	LAC2	---	---	---	---	0.53	0.47	0	---	0	0.00
APAP-LAC2-MCC1-CCS2-MgSt2- blend 1	APAP	LAC2	MCC1	---	CCS2	MgSt2	0.1	0.655	0.2	---	0.035	0.01
APAP-LAC2-MCC1-CCS2-MgSt2- blend 2	APAP	LAC2	MCC1	---	CCS2	MgSt2	0.2	0.555	0.2	---	0.035	0.01
APAP-LAC2-MCC1-CCS2-MgSt2- blend 3	APAP	LAC2	MCC1	---	CCS2	MgSt2	0.4	0.355	0.2	---	0.035	0.01
IBU-LAC2- blend 1	IBU	LAC2	---	---	---	---	0.066	0.934	0	---	0	0.00
IBU-LAC2- blend 2	IBU	LAC2	---	---	---	---	0.265	0.735	0	---	0	0.00
IBU-LAC2- blend 3	IBU	LAC2	---	---	---	---	0.53	0.47	0	---	0	0.00
IBU-LAC2-MCC1-CCS2-MgSt2- blend 1	IBU	LAC2	MCC1	---	CCS2	MgSt2	0.05	0.705	0.2	---	0.035	0.01
IBU-LAC2-MCC1-CCS2-MgSt2- blend 2	IBU	LAC2	MCC1	---	CCS2	MgSt2	0.2	0.555	0.2	---	0.035	0.01
IBU-LAC2-MCC1-CCS2-MgSt2- blend 3	IBU	LAC2	MCC1	---	CCS2	MgSt2	0.4	0.355	0.2	---	0.035	0.01
MFA-LAC2- blend 1	MFA	LAC2	---	---	---	---	0.066	0.934	0	---	0	0

MFA-LAC2- blend 2	MFA	LAC2	---	---	---	---	0.265	0.735	0	---	0	0
MFA-LAC2- blend 3	MFA	LAC2	---	---	---	---	0.464	0.536	0	---	0	0
MFA-LAC2-MCC1-CCS2-MgSt2- blend 1	MFA	LAC2	MCC1	---	CCS2	MgSt2	0.05	0.705	0.2	---	0.035	0.01
MFA-LAC2-MCC1-CCS2-MgSt2- blend 2	MFA	LAC2	MCC1	---	CCS2	MgSt2	0.2	0.555	0.2	---	0.035	0.01
MFA-LAC2-MCC1-CCS2-MgSt2- blend 3	MFA	LAC2	MCC1	---	CCS2	MgSt2	0.35	0.405	0.2	---	0.035	0.01
APAP-LAC2- blend 1	APAP	LAC2	---	---	---	---	0.066	0.934	0	---	0	0
APAP-LAC2- blend 2	APAP	LAC2	---	---	---	---	0.133	0.868	0	---	0	0
APAP-LAC2- blend 3	APAP	LAC2	---	---	---	---	0.199	0.801	0	---	0	0
APAP-LAC2-MCC1-CCS2-MgSt2- blend 1	APAP	LAC2	MCC1	---	CCS2	MgSt2	0.05	0.705	0.2	---	0.035	0.01
APAP-LAC2-MCC1-CCS2-MgSt2- blend 2	APAP	LAC2	MCC1	---	CCS2	MgSt2	0.1	0.655	0.2	---	0.035	0.01
APAP-LAC2-MCC1-CCS2-MgSt2- blend 3	APAP	LAC2	MCC1	---	CCS2	MgSt2	0.15	0.605	0.2	---	0.035	0.01
APAP-MCC1-DCPA-CCS1-MgSt1- blend 1	APAP	MCC1	DCPA	---	CCS1	MgSt1	0.01	0.48	0.48	---	0.02	0.01
APAP-MCC1-DCPA-CCS1-MgSt1- blend 2	APAP	MCC1	DCPA	---	CCS1	MgSt1	0.2	0.385	0.385	---	0.02	0.01
APAP-MCC1-DCPA-CCS1-MgSt1- blend 3	APAP	MCC1	DCPA	---	CCS1	MgSt1	0.4	0.285	0.285	---	0.02	0.01
APAP-MCC1-DCPA-CCS1-MgSt1- blend 4	APAP	MCC1	DCPA	---	CCS1	MgSt1	0.01	0.465	0.465	---	0.05	0.01
APAP-MCC1-DCPA-CCS1-MgSt1- blend 5	APAP	MCC1	DCPA	---	CCS1	MgSt1	0.2	0.37	0.37	---	0.05	0.01
APAP-MCC1-DCPA-CCS1-MgSt1- blend 6	APAP	MCC1	DCPA	---	CCS1	MgSt1	0.4	0.27	0.27	---	0.05	0.01
APAP-MCC1-DCPA-CCS1-MgSt1- blend 7	APAP	MCC1	DCPA	---	CCS1	MgSt1	0.01	0.45	0.45	---	0.08	0.01
APAP-MCC1-DCPA-CCS1-MgSt1- blend 8	APAP	MCC1	DCPA	---	CCS1	MgSt1	0.2	0.355	0.355	---	0.08	0.01
APAP-MCC1-DCPA-CCS1-MgSt1- blend 9	APAP	MCC1	DCPA	---	CCS1	MgSt1	0.4	0.255	0.255	---	0.08	0.01
APAP-MCC1-MAN-CCS1-MgSt1- blend 1	APAP	MCC1	MAN	---	CCS1	MgSt1	0.01	0.48	0.48	---	0.02	0.01
APAP-MCC1-MAN-CCS1-MgSt1- blend 2	APAP	MCC1	MAN	---	CCS1	MgSt1	0.2	0.385	0.385	---	0.02	0.01
APAP-MCC1-MAN-CCS1-MgSt1- blend 3	APAP	MCC1	MAN	---	CCS1	MgSt1	0.4	0.285	0.285	---	0.02	0.01

XXX-LAC2-MCC2--CCS2-MgSt2- blend 1	---	LAC2	MCC2	---	CCS2	MgSt2	0	0.755	0.2	---	0.035	0.01
XXX-LAC2-MCC2-CCS2-MgSt2- blend 2	---	LAC2	MCC2	---	CCS2	MgSt2	0	0.805	0.15	---	0.035	0.01
XXX-LAC2-MCC2-CCS2-MgSt2- blend 3	---	LAC2	MCC2	---	CCS2	MgSt2	0	0.4775	0.4755	---	0.035	0.01
XXX-LAC2-MCC2-CCS2-MgSt2- blend 4	---	LAC2	MCC2	---	CCS2	MgSt2	0	0.205	0.75	---	0.035	0.01
XXX-LAC2-MCC2-CCS2-MgSt2- blend 5	---	LAC2	MCC2	---	CCS2	MgSt2	0	0.155	0.8	---	0.035	0.01
XXX-LAC2-MCC2-CCS2-MgSt2- blend 6	---	LAC2	MCC2	---	CCS2	MgSt2	0	0.105	0.85	---	0.035	0.01
XXX-LAC2-MCC2-CCS2-MgSt2- blend 7	---	LAC2	MCC2	---	CCS2	MgSt2	0	0	0.955	---	0.035	0.01
GRF-MCC1-LAC1-CCS1-MgSt1- blend 1	GRF	MCC1	LAC1	---	CCS1	MgSt1	0.01	0.678	0.097	---	0.05	0.01
GRF-MCC1-LAC1-MgSt1- blend 1	GRF	MCC1	---	---	CCS1	MgSt1	0.01	0.465	0.465	---	0.05	0.01
GRF-MCC1-LAC1-CCS1-MgSt1- blend 2	GRF	MCC1	LAC1	---	CCS1	MgSt1	0.05	0.93	0	---	0.05	0.01
GRF-MCC1-LAC1-MgSt1- blend 2	GRF	MCC1	---	---	CCS1	MgSt1	0.05	0.445	0.445	---	0.05	0.01
GRF-MCC1-LAC1-CCS1-MgSt1- blend 3	GRF	MCC1	LAC1	---	CCS1	MgSt1	0.10	0.89	0	---	0.05	0.01
GRF-MCC1-LAC1-MgSt1- blend 3	GRF	MCC1	---	---	CCS1	MgSt1	0.10	0.42	0.42	---	0.05	0.01
GRF-MCC1-LAC1-CCS1-MgSt1- blend 4	GRF	MCC1	LAC1	---	CCS1	MgSt1	0.15	0.84	0	---	0.05	0.01
GRF-MCC1-LAC1-MgSt1- blend 4	GRF	MCC1	---	---	CCS1	MgSt1	0.15	0.395	0.395	---	0.05	0.01
GRF-MCC1-LAC1-CCS1-MgSt1- blend 5	GRF	MCC1	LAC1	---	CCS1	MgSt1	0.20	0.79	0	---	0.05	0.01
GRF-MCC1-LAC1-MgSt1- blend 5	GRF	MCC1	---	---	CCS1	MgSt1	0.20	0.37	0.37	---	0.05	0.01
GRF-MCC1-LAC1-CCS1-MgSt1- blend 6	GRF	MCC1	LAC1	---	CCS1	MgSt1	0.30	0.74	0	---	0.05	0.01
GRF-MCC1-LAC1-MgSt1- blend 6	GRF	MCC1	---	---	CCS1	MgSt1	0.30	0.32	0.32	---	0.05	0.01
GRF-MCC1-LAC1-CCS1-MgSt1- blend 7	GRF	MCC1	LAC1	---	CCS1	MgSt1	0.40	0.64	0	---	0.05	0.01
GRF-MCC1-LAC1-MgSt1- blend 7	GRF	MCC1	---	---	CCS1	MgSt1	0.40	0.27	0.27	---	0.05	0.01

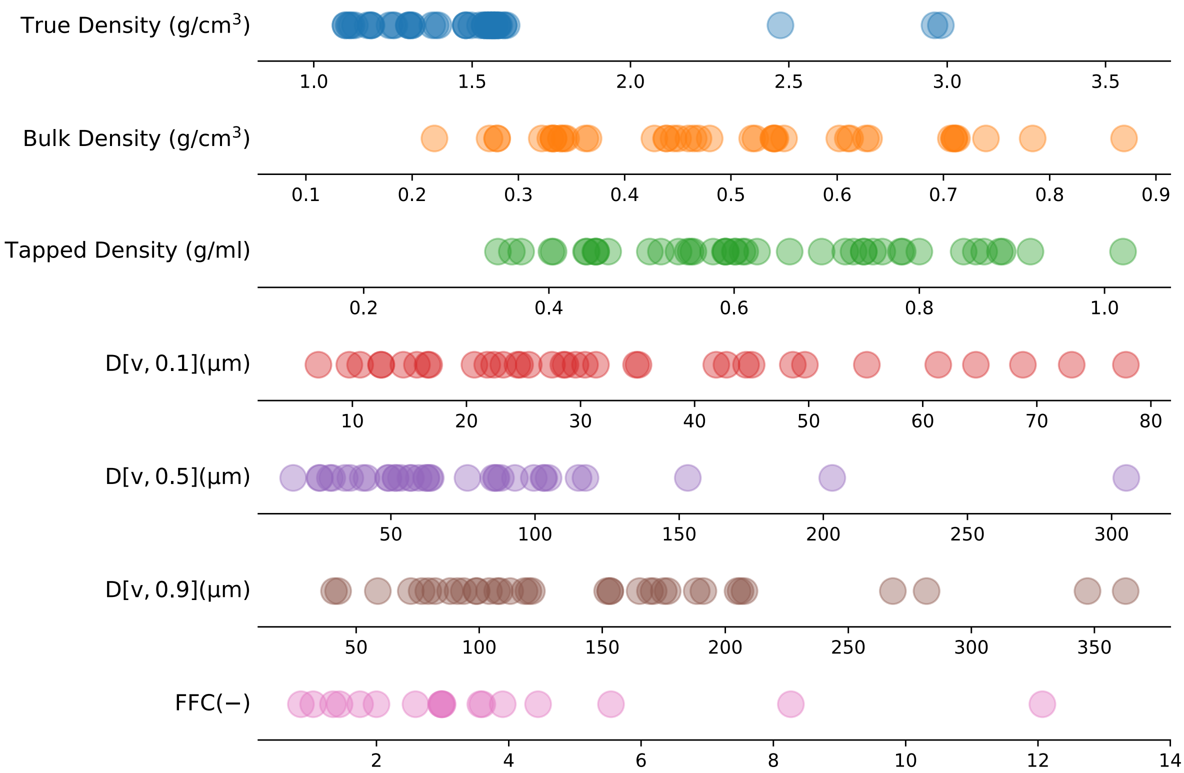

Figure S1: Visual summary of the range of raw material properties. $D[v, 0.1]$, $D[v, 0.5]$, and $D[v, 0.9]$ denote 10%, 50% and 90% percentiles of the volume-based frequency distribution of particle size.

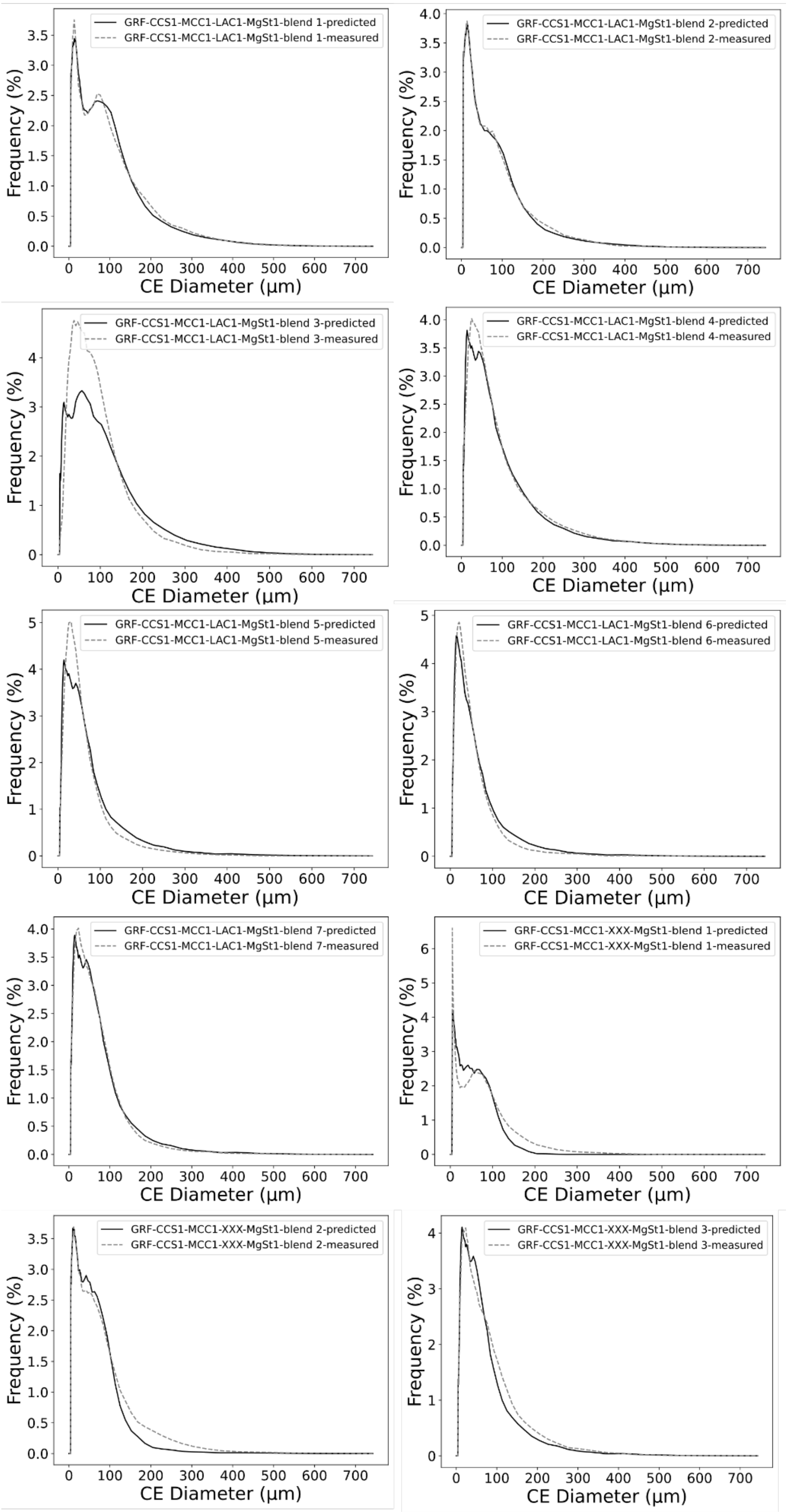

Figure S2: Number-based PSD of mixtures predicted by the probabilistic model vs. experimental measurements. CE diameter denotes the circular equivalent diameter. The “XXX” in the blend name denotes no API or no second excipient.

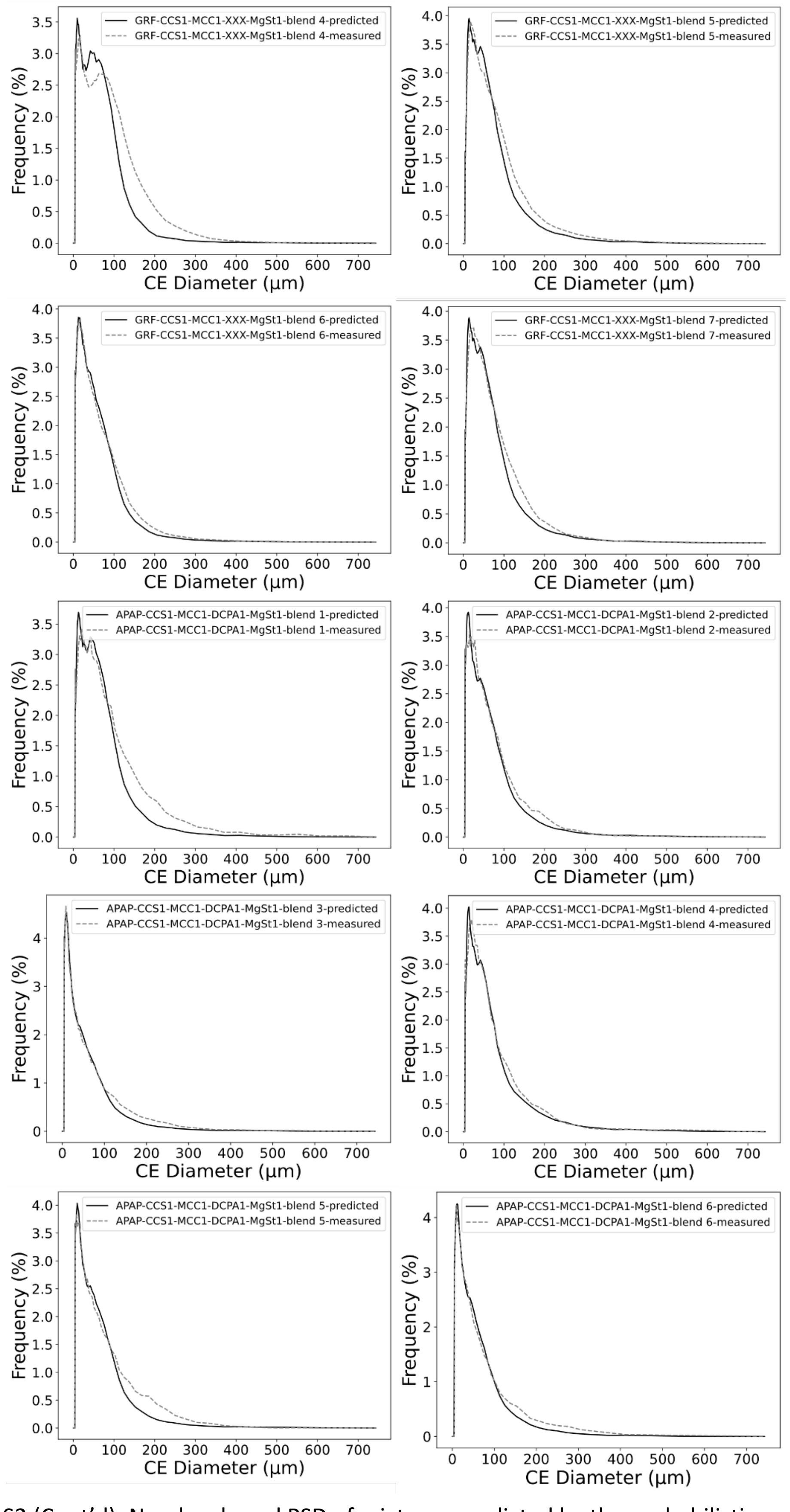

Figure S2 (Cont'd): Number-based PSD of mixtures predicted by the probabilistic model vs. experimental measurements. CE diameter denotes the circular equivalent diameter. The "XXX" in the blend name denotes no API or no second excipient.

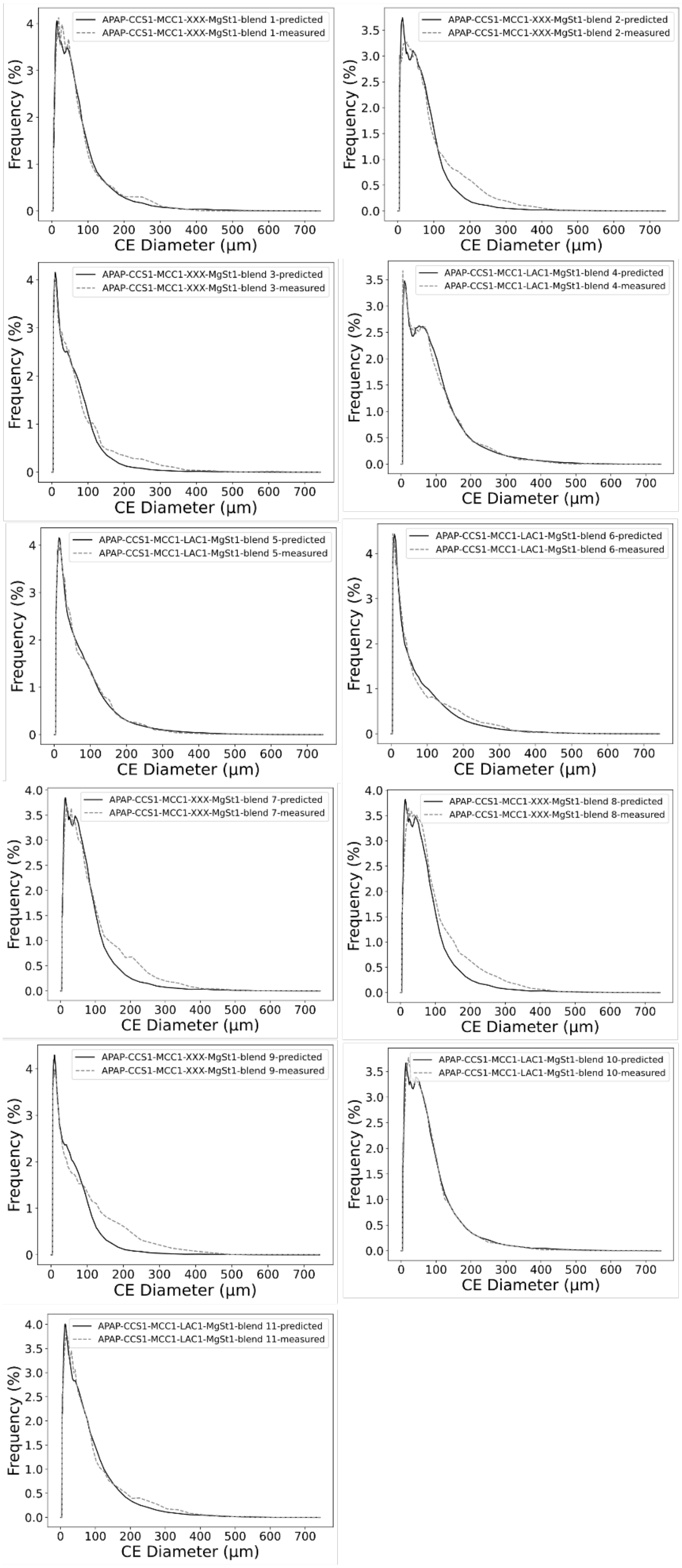

Figure S2 (Cont'd): Number-based PSD of mixtures predicted by the probabilistic model vs. experimental measurements. CE diameter denotes the circular equivalent diameter. The "XXX" in the blend name denotes no API or no second excipient.

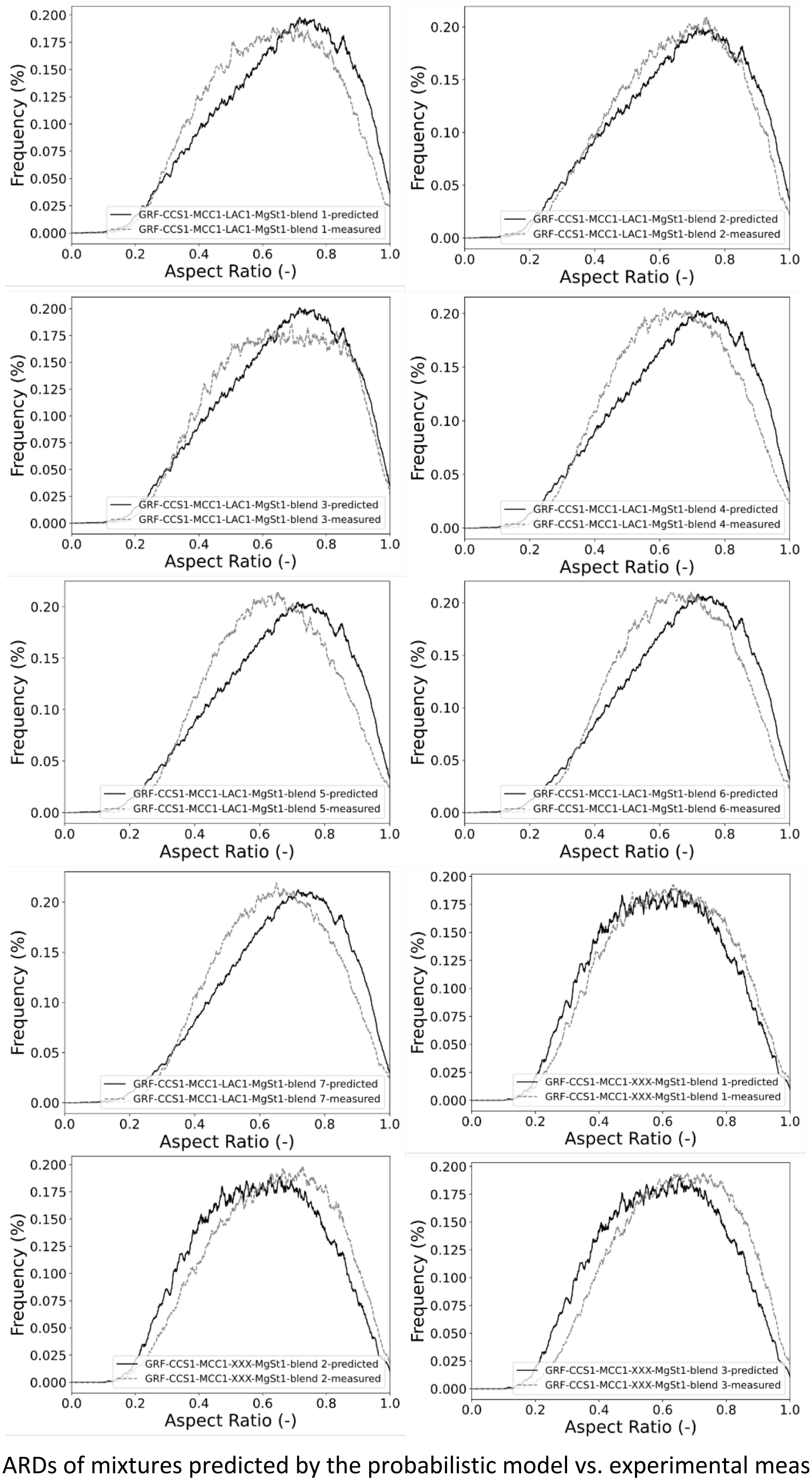

Figure S3: ARDs of mixtures predicted by the probabilistic model vs. experimental measurements. The “XXX” in the blend name denotes no API or no second excipient.

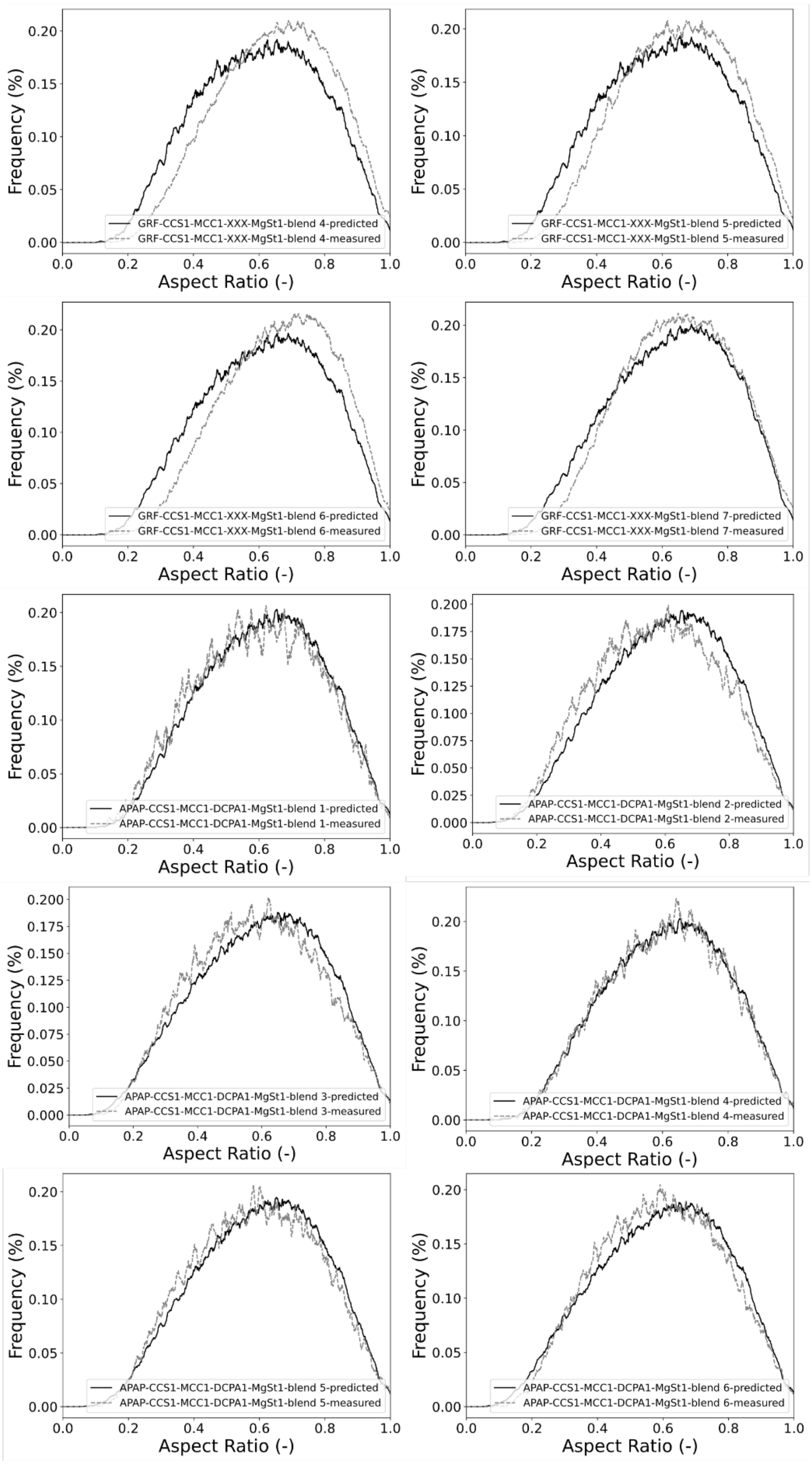

Figure S3 (Cont'd): ARDs of mixtures predicted by the probabilistic model vs. experimental measurements. The "XXX" in the blend name denotes no API or no second excipient.

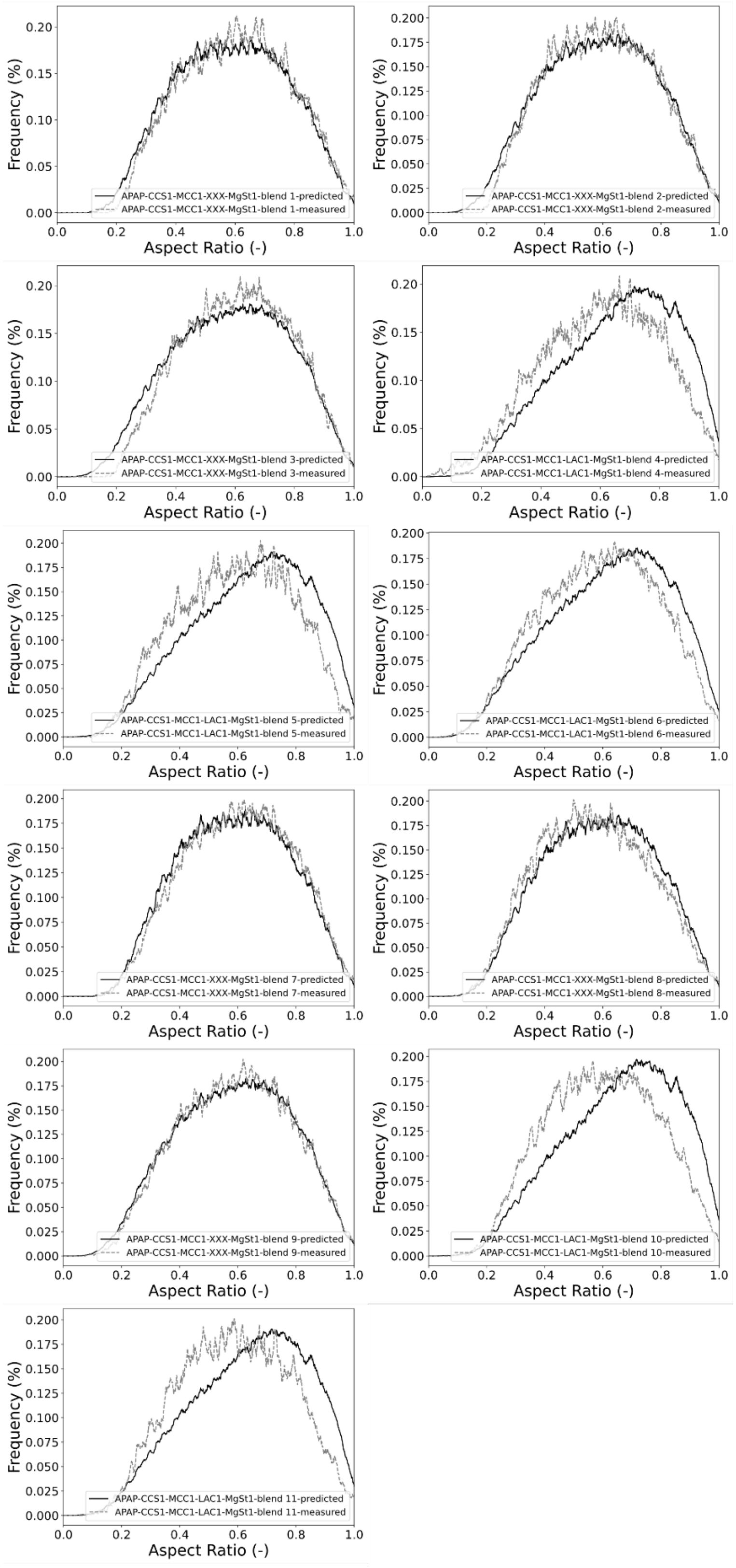

Figure S3 (Cont'd): ARDs of mixtures predicted by the probabilistic model vs. experimental measurements. The "XXX" in the blend name denotes no API or no second excipient.

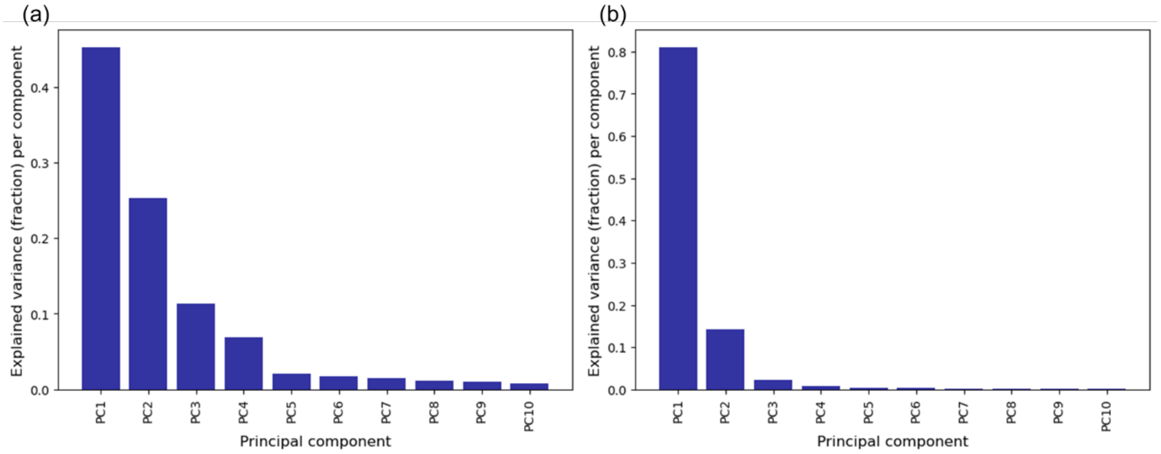

Figure S4: Explained variance of first ten components in PCA models applied to (a) PSDs and (b) ARDs of mixtures.

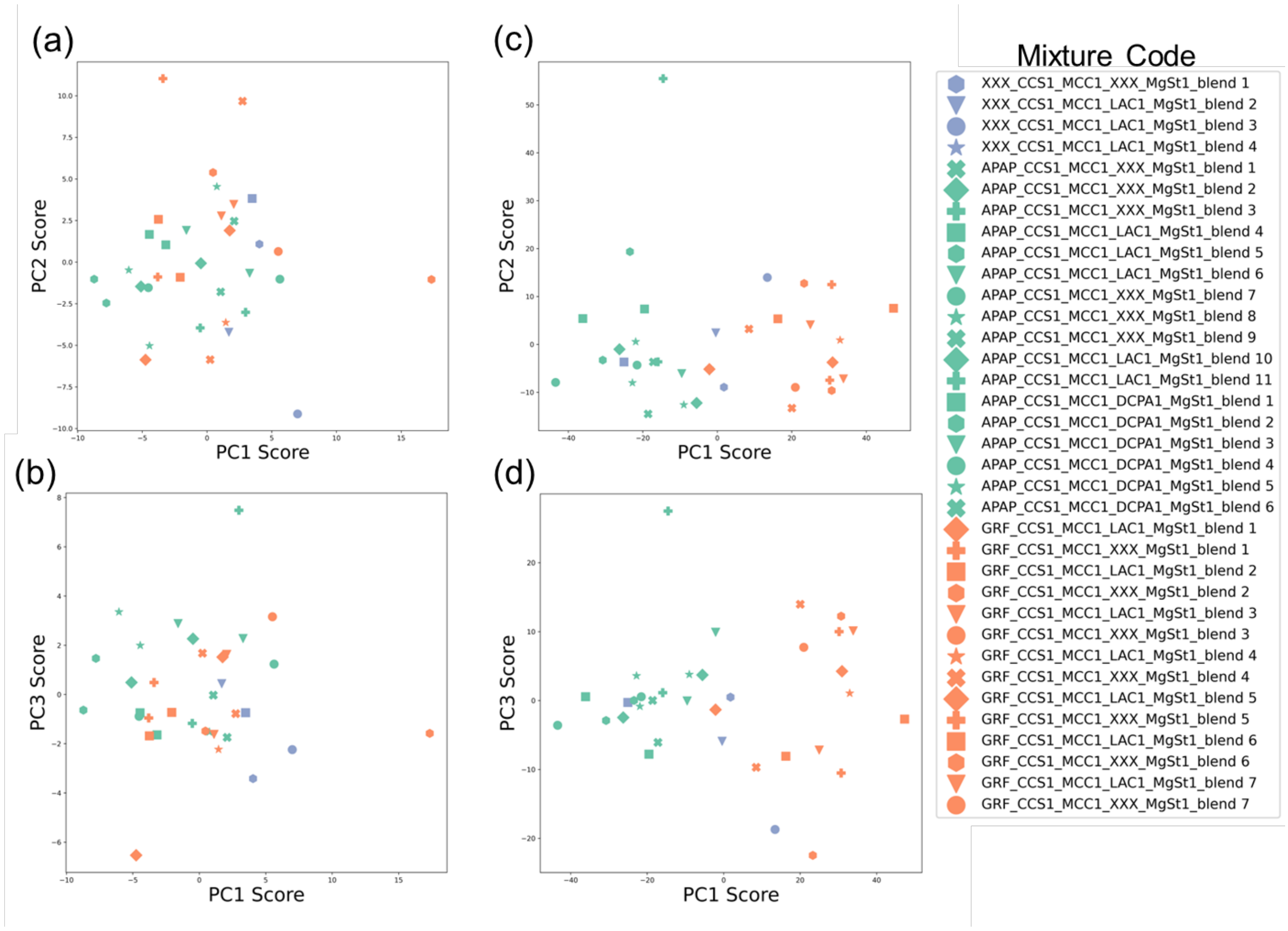

Figure S5: First three principal scores of PSDs (a, b) and ARDs (c, d) of mixtures. The “XXX” in the blend name denotes no API or no second excipient.

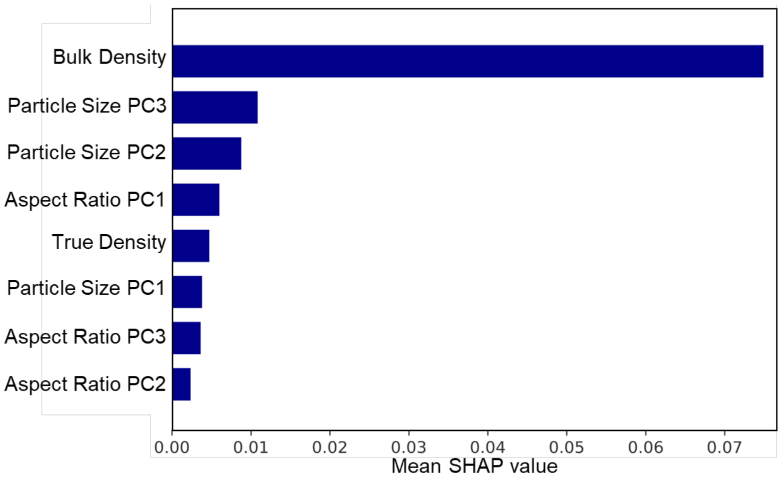

Figure S6: Feature importance analysis for the XGBoost tapped density mixture model. The features are ranked based on their mean SHAP value.

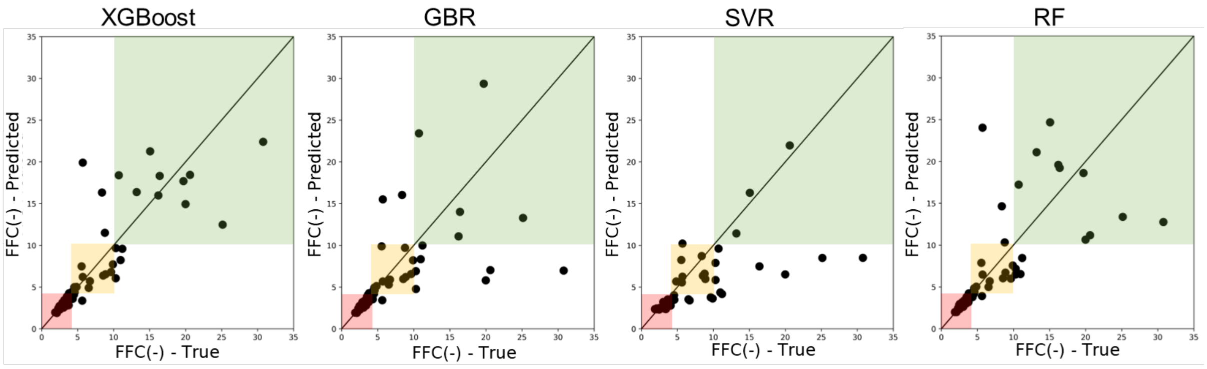

Figure S7: Predicted mixture FFC using four data-driven models vs. true (experimental) measurements of blends. The green, yellow, and red regions are highlighted to demonstrate the classification of free-flowing ($FFC > 10$), easy-flowing ($4 < FFC \leq 10$), and cohesive powders ($FFC \leq 4$), respectively.

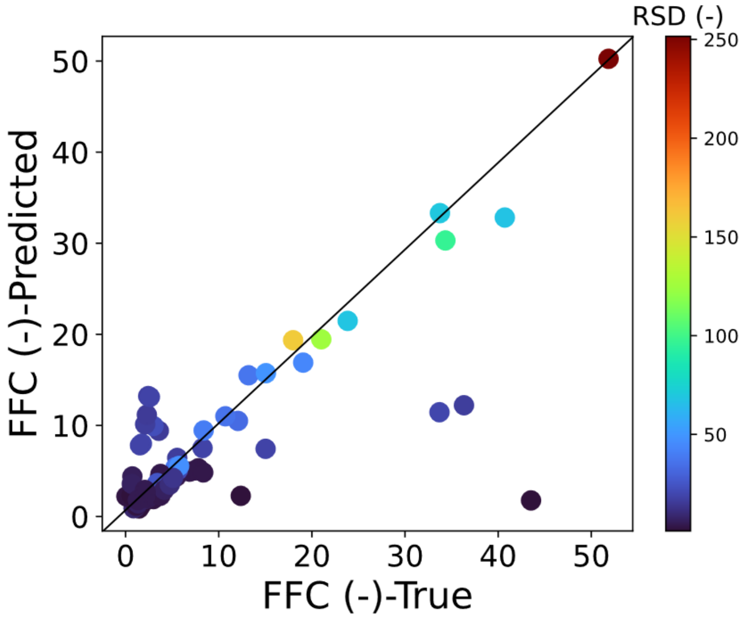

Figure S8: Prediction performance of RF model and the associated uncertainty in predicted FFC values of raw material components. The colour bar shows the RSD of the predicted data points.
